# Antarctic Krill 454 Pyrosequencing Reveals Chaperone and Stress Transcriptome

**DOI:** 10.1371/journal.pone.0015919

**Published:** 2011-01-06

**Authors:** Melody S. Clark, Michael A. S. Thorne, Jean-Yves Toullec, Yan Meng, Le Luo Guan, Lloyd S. Peck, Stephen Moore

**Affiliations:** 1 British Antarctic Survey, Natural Environment Research Council, Cambridge, United Kingdom; 2 UPMC Université Paris 06, UMR 7144 CNRS, Adaptation et Diversité en Milieu Marin, Station Biologique de Roscoff, Roscoff, France; 3 Department of Agricultural, Food and Nutritional Science, University of Alberta, Edmonton, Canada; Argonne National Laboratory, United States of America

## Abstract

**Background:**

The Antarctic krill *Euphausia superba* is a keystone species in the Antarctic food chain. Not only is it a significant grazer of phytoplankton, but it is also a major food item for charismatic megafauna such as whales and seals and an important Southern Ocean fisheries crop. Ecological data suggest that this species is being affected by climate change and this will have considerable consequences for the balance of the Southern Ocean ecosystem. Hence, understanding how this organism functions is a priority area and will provide fundamental data for life history studies, energy budget calculations and food web models.

**Methodology/Principal Findings:**

The assembly of the 454 transcriptome of *E. superba* resulted in 22,177 contigs with an average size of 492bp (ranging between 137 and 8515bp). In depth analysis of the data revealed an extensive catalogue of the cellular chaperone systems and the major antioxidant proteins. Full length sequences were characterised for the chaperones HSP70, HSP90 and the super-oxide dismutase antioxidants, with the discovery of potentially novel duplications of these genes. The sequence data contained 41,470 microsatellites and 17,776 Single Nucleotide Polymorphisms (SNPs/INDELS), providing a resource for population and also gene function studies.

**Conclusions:**

This paper details the first 454 generated data for a pelagic Antarctic species or any pelagic crustacean globally. The classical “stress proteins”, such as HSP70, HSP90, ferritin and GST were all highly expressed. These genes were shown to be over expressed in the transcriptomes of Antarctic notothenioid fish and hypothesized as adaptations to living in the cold, with the associated problems of decreased protein folding efficiency and increased vulnerability to damage by reactive oxygen species. Hence, these data will provide a major resource for future physiological work on krill, but in particular a suite of “stress” genes for studies understanding marine ectotherms' capacities to cope with environmental change.

## Introduction

The Southern Ocean is an important breeding and/or foraging location for a wide range of charismatic megafauna such as whales, seals, penguins and other sea birds including albatrosses. Whilst these species fascinate the public, they represent the apex of a complex food chain, the keystone species of which is the Antarctic krill *Euphausia superba* [1]. This shrimp-like crustacean is not only a major prey item for these animals, but is also a significant consumer, grazing on the phytoplankton bloom in the austral summer and on algae under the sea ice in winter. In a more global context, it has been suggested to be the most abundant eukaryotic species in the World's Oceans [2] existing in large schools or swarms with densities that may be between 10,000–30,000 individual animals per square metre [3], [4]. Current catches are 125,000–150, 000 tonnes per year [5], but at its peak in 1982, krill fisheries comprised 13% of the global annual catches of crustaceans [6]. Hence, *E. superba* with a circumpolar distribution plays not only a pivotal role in the Antarctic ecosystem, but also significantly impacts on the economy of the Southern Ocean fishing industry [1], [7].

Research on krill has a long history, reaching back to the “Discovery” expeditions of the 1920/1930s that sought to understand the drivers of fluctuating populations of baleen whales which fed on krill and which were then harvested for their oil [8]. More recently, research efforts have also focussed on stock estimates and distributions, again with regard to fisheries exploitation [7]. Whilst catches have remained relatively stable and never reached the maximum TAC (total allowable catch) defined by the regulatory body the Commission for the Conservation of Antarctic Marine Living Resources (CCAMLR), there is growing concern about decreasing krill stocks and any consequential effects on both the Southern Ocean ecosystem and the fisheries industry [9].

An historical analysis of krill numbers in the southwest Atlantic sector (which contains over half the total krill stocks of the Southern Ocean) [10], [11] indicates that there has been a significant decline in population densities since the 1970's which correlates with the extent of winter sea ice cover. The ice provides an essential habitat and food supply via ice algae for these animals over the winter period [10], [12]. The southwest Atlantic sector of the Southern Ocean includes the Antarctic Peninsula, which is experiencing some of the most rapid rises in sea water temperature on the planet [13] with a 1°C increase in the surface layers of the Bellingshausen Sea in 50 years [14], [15] and reduced winter sea ice duration [16]. This continued and rapid temperature rise has profound implications, not only for krill stocks, but also for broader food web interactions in the Southern Ocean [10], [17]–[22] and for the fishing industry [7], [9], [23].

There is a considerable body of scientific knowledge about the general biology and ecology of krill, in particular growth, biochemical composition and reproduction. One of the main drivers for understanding krill biology has been the fisheries' requirements for stock forecasting and conservation measures, but this is now joined by concerns over climate change effects and the requirement to take a more holistic over-view to understand food web structures [7], [24], [25]. However, there is very little knowledge of processes at the tissue, cellular or molecular level. As a consequence, and given the pivotal role of this key species in the Southern Ocean ecosystem, detailed physiological and molecular studies are critical if we are to understand this species and its responses to a changing environment. Such studies would provide fundamental data for a deeper understanding of life histories, as well as important parameters for energy budget calculations and models [26].

Working with wild free living krill is beyond the capability of current technologies. Therefore cellular, tissue and molecular data can only be derived from either individuals recently sampled from the sea, with the concommitant stresses involved or from laboratory manipulations of aquarium held animal stocks. Hence a fundamental issue for the future progression of these studies will be the linking of laboratory developed assays with wild caught sampling [27], [28]. The physiology of *E. superba* has been studied in a number of laboratory based experiments for periods of up to 5 months using non-specialised facilities [25], [26], [29]. Some of these studies have highlighted the diversity of problems encountered when working on a pelagic species, such as krill adapted to living in swarms with social aggregations [29], [30]. These studies have emphasized the necessity of understanding stress responses in this species as an *a priori* to improve experimental protocol and also to provide a baseline laboratory physiological metric for such comparative field sampling [26], [30] as such molecular analyses can provide a more detailed description of animal condition over observational physiology approaches [31]. To date there are only 3095 ESTs from *E. superba* in the public databases, but the classical “stress genes” are largely absent from these gene sets [32], [33].

Here we describe the transcriptome of *E. superba*, the first 454 generated data for a pelagic Antarctic species or any pelagic crustacean globally. Analysis here focuses on chaperone genes, in particular the typical “stress” genes of the Heat Shock Protein (HSP) family. This data will provide a major resource for future physiological work on krill, but in particular a suite of “stress” genes for studies understanding marine ectotherms capacities to cope with environmental change, such as future elevated sea water temperatures, ocean acidification or water freshening etc.

## Results and Discussion

The non-normalised krill libraries were subjected to a full 454 run that yielded 943,817 reads. After cleaning the data and removing small reads, 699,248 reads containing 205,888,141 bases with an average read size of 293 bp were entered into Newbler [34] for assembly. These assembled into the 22,177 contigs (261,280 reads) which were used for the further analysis. Because the aim of this project was to conduct a preliminary characterisation of the krill transcriptome, with, as described below, an emphasis on chaperones and stress-related genes for future analyses there was a requirement for longer sequences of good quality which would enable us to distinguish between gene family members. Hence the descriptive analysis presented here utilised only the contigs produced by the assembly. Whilst the singletons potentially contain useful lowly expressed sequences, they also contain a substantial proportion of artefacts derived from cDNA synthesis, sequencing and contamination [35]. However these will be retained within our databases and utilised when more targeted pyrosquencing experiments (using both 454 and short reads) will generate an enlarged transcriptome. The contigs ranged in size from 137 bp to 8515 bp, with an average size of 491.9 bp. 48 contigs were greater than 3kb and 94 contigs comprised more than 300 reads, with the largest contig of 8515 bp comprising the most reads with 740 sequences. Self BLAST of this dataset produced only 625 matches with a value of e^−100^, indicating a low level (<3%) of redundancy in the assembly of the reads. The contigs contained 41,470 repeat sequence motifs in coding and/or UTR regions (microsatellites), of which 339 comprised over 7 exact repeat units (Table S1). There were 27,776 SNPs/INDELS present in 3,980 contigs designated as high confidence by the Newbler program (Table S2), although a further circa 25,000 SNPs were identified at lower confidence level as defined by Newbler [34] Hence these provide significant resources for researchers in allied fields and will fuel investigations into, for example, population structure and gene flow dynamics in this crucial pelagic species.

BLAST sequencing similarity searching of the GenBank non-redundant database produced matches against 5563 of the contigs using a <1e^−10^ cut off value (representing 25% of the total number of contigs). Although this seems rather low, the number of matches is higher when compared to those of other non-model marine invertebrates which have recently been subjected to 454 transcriptome analyses (c.f. 12% in the blue mussel *Mytilus galloprovincialis*, 17% in the Antarctic bivalve *Laternula elliptica* and 23.9% in larvae of the coral *Acropora millipora* [35]–[37]. This is due to the phylogenetic position of *E. superba* within the *Arthropoda*, a phylum which contains a number of species that have been subjected to whole genome sequencing programmes. The prime example of these is the fruit fly *Drosophila melanogaster* and the insect vectors of human disease such as *Anopheles gambiae* (www.vectorbase.org). Of these, the *Drosophila* genes, in particular, are well annotated (www.flybase.org). However, *E. superba* is a marine invertebrate and more closely related (within the sub-phylum *Eucrustacea*, super-order *Eucarida*) are a number of commercially important seafood species, such as the Pacific white shrimp *Litopenaeus vannamei*, the black tiger shrimp *Penaeus monodon* and the fleshy prawn *Fenneropenaeus chinensis*. These have been subjected to several medium-scale EST projects in the past and comprise the majority of the 33,813 nucleotide entries and 400,793 ESTs for the *Eucarida* in the public database (www.ncbi.nlm.nih.gov) (as of 15/05/10). BLAST sequence similarity searches against these more morphologically and physiologically similar species produces a greater number of matches (35–43%) (Table 1) and some of these, particularly the immune-related genes, are well annotated and characterised [c.f. 38]–[42]. To date *E.superba* is poorly represented in the database with only 3095 ESTs [32]–[33], of which over 80% were represented in the transcriptome presented here (Table 1). The *E. superba* mitochondrial genome has been almost completely sequenced on two occasions with the aim of producing markers for exploitation and management of krill fisheries resources [43], [44]. Whilst a previous 454 run on a non-model species, *Mytilus galloprovincialis* produced enough mitochondrial reads to enable alignment of this new data against the relevant mitochondrial sequence [36], this was not possible on the krill data, as only 8 mitochondrial genes (cytochrome c oxidase, subunits 1, 2 and 3, ATP synthase subunit 6 and NADH dehydrogenase subunits 1,2,4 and 5) were present in our dataset, which may be due to the fact that non-normalised libraries were produced from a single source of krill material. Sequencing of the transcriptomes of krill subjected to different treatments will almost certainly enhance the identification of mitochondrial genes.

**Table 1:** Representation of *Eucarida* species in the public databases (at 15/07/10).

Species	No of ESTs in database	BLASTn matches			tBLASTx matches		
		krill 454	ESTs	% match	krill 454	ESTs	% match
*Euphausia superba*	3095	2177	2567	83	2560	2547	82
*Fenneropenaeus chinensis*	10511	556	1706	16	1759	3717	35
*Penaeus monodon*	35690	968	10464	29	3141	15400	43
*Litopenaeus vannamei*	162755	1412	24482	15	4893	63145	39

Number of species-specific ESTS and the number of krill ESTs that match a corresponding number of *Eucarida* ESTs.

To obtain an overall view of the krill transcriptome and also as part of the contig verification process, all contigs comprising over 450 reads were analysed in-depth. These most commonly expressed sequences with an associated database match represented a varied mix of functional groups (Table 2). The largest contig, with a length of 8515bp also contained the highest number of reads (740). BLAST sequence similarity searching of this contig produced a match with an E-value of 0.0 against the shrimp, *Litopenaeus vannemei* β-1,3-glucan binding protein (βGBP-HDL) [39] (Table 2). The full length of this 1454 amino acid protein was represented in the krill contig. Not surprisingly, given the E-value, there was strong conservation at the amino acid level with 41.2% identity and 59.4% similarity between the two putative proteins. There was also conservation of the precursor N-terminal domain and processing sites for the mature peptide. However, out of the 4 O-glycosylation and 5 N-glycosylation sites found to be preserved between the two Penaeid shrimp and crayfish βGBP-HDL proteins, only 2 and 1 respectively were present in the krill sequence, indicating potential differences in post translational modification [39]. Functional characterisation of the shrimp protein revealed a dual function: binding of β-glucans trigger the activation of this pattern-recognition protein and the prophenol-oxidase system, which is a central component of the shrimp innate immunity defence system, but βGBP-HDL is also involved in lipid transport [45], [46]. Whilst the definition as to which of these functions predominates is not possible within the confines of this descriptive study, the second most common sequence was fatty acid synthase, a key enzyme in the production of fatty acids. The Antarctic environment is subject to extreme seasonality with huge phytoplankton blooms during the austral summer [47]–[49] and strong seasonal physiology is known for many Antarctic species [50], [51]. The krill samples used for this transcriptome were caught during the summer and hence during the period when the animals were taking advantage of the ready food supply to fuel the seasonal increase in metabolism, growth and reproduction [52], [53]. This latter process is also supported by the presence of vitellogenin, a major reproductive protein.

**Table 2:** Most commonly expressed sequences with associated BLAST matches.

Contig ID	Length (bp)	No of reads	Description	Species	Common name	E-value
00790	8515	740	β-1,3-glucan binding protein	*Litopenaeus vannemei*	Pacific white shrimp	0.0
00604	7491	689	Fatty acid synthase	*Aedes aegypti*	Yellow fever mosquito	0.0
00865	5235	643	Myosin heavy chain	*Aedes aegypti*	Yellow fever mosquito	6.4 e-253
01872	2994	617	Ribonucleoside diphosphate reductase	*Aedes aegypti*	Yellow Fever mosquito	0.0
00704	462	596	Ribosomal protein L10	*Callinectes sapidus*	Blue crab	1.0 e-70
00239	279	576	Myosin light chain	*Litopenaeus vannemei*	Pacific white shrimp	3.0 e-16
00111	2061	568	ATP synthase	*Bombyx mori*	Silk worm	0.0
02321	2341	534	Eukaryotic translation initiation factor 5A	*Penaeus monodon*	Black tiger prawn	9.0 e-63
00248	1639	525	NADH dehydrogenase sub-unit 4	*Euphausia superba*	Antarctic krill	0.0
00826	1750	502	Calreticulin	*Fenneropenaeus chinensis*	Fleshy prawn	1.5 e-152
00709	2128	499	Elongation factor 1γ	*Artemia salina*	Brine shrimp	1.4 e-152
01202	4840	493	Vigilin	*Culex quinquefasciatus*	mosquito	3.8 e-257
00995	505	492	60s ribosomal protein L5	*Ixodes scapularis*	Blacklegged tick	1.0 e-45
20909	849	490	Dehydrogenase, glyceraldehyde dephosphate	*Pleocyemata sp.*	Lobster	1.5 e-122
19460	1984	484	Chaperonin	*Nasonia vitripennis*	Jewel wasp	0.0
00477	800	483	Vitellogenin	*Pandalopsis japonica*	Shrimp species	4.9 e-17
00056	867	483	Ferritin peptide	*Fenneropenaeus chinensis*	Fleshy prawn	4.7 e-71
00581	860	479	ATP dependant RNA helicase	*Caligus rogercresseyi*	Sea louse	3.1 e-181
21501	2124	474	Cyclin A	*Penaeus monodon*	Black tiger prawn	4.7 e-155
18212	417	464	Ribosomal protein L1	*Lonomia obliqua*	Caterpillar	1.4 e-40
19379	1095	458	Death-associated protein-like	*Penaeus monodon*	Giant tiger prawn	3.2 e-16
00914	1918	456	Protein disuphide isomerase	*Scylla paramamosain*	Green mud crab	1.4 e-162
01103	1039	455	Acidic ribosomal protein P0	*Spodoptera frugiperda*	Fall armyworm	4.0 e-115

The majority of the remainder of the putative genes were clearly related to this seasonal physiology and an active metabolism with presence of transcripts involved in DNA replication and RNA metabolism (ribonucleoside diphosphate reductase and vigilin respectively), protein synthesis (elongation factors and ribosomal genes), energy production (dehydrogenase, glyceraldehyde dephosphatase), cell division (cyclin A) and growth (myosin heavy and light chains). The relatively high levels of vigilin may potentially be linked to those of vitellogenin, as although it is suggested to play a general role in RNA metabolism [54], it has been specifically shown to stabilise vitellogenin transcripts in some species [55]. The seemingly odd presence of an apoptosis-related gene (contig19379) is almost certainly linked to the processes involved in ecdysis and moulting. Again, this is a process with increased activity in the austral summer, being linked to nutritional status and environmental conditions [56]. Overall, this initial survey of the pyrosequencing data substantiates a previous molecular study, which indicated that krill sampled in the summer were far more active than those sampled in winter [33].

However, of most interest to our research, was the presence of genes involved in chaperone functions and the stress response in the most commonly expressed sequences (calreticulin, chaperonin, protein disulphide isomerase and ferritin) (Table 2). Also, whilst not in this listing, the heat shock proteins HSP90 and HSP70 were also highly represented in the transcriptome with 417 and 307 reads respectively. To futher understand these systems and provide a baseline for future studies on the environmental stress response of *E. superba*, a number of putative genes were examined in more detail. These sequences were identified from the BLAST sequence similarity results as being involved in either chaperone or antioxidant functions. Where possible, the more commonly known names of the chaperone genes have also been linked to the new nomenclature as designated by the HUGO Gene Nomenclature committee and NCBI [57].

### The cellular chaperone systems

The cellular chaperones can be divided into two main categories: the cytosolic and the endoplasmic reticulum (ER) systems. Many of these proteins are expressed ubiquitously in the normal cell state to aid in the folding of native polypeptides and their translocation to different cellular compartments [58], [59]. However, during the stress response, they may be up-regulated to further assist mis-folded proteins to attain or regain their native states and also target degraded proteins and regulate their removal from the cell, thus preventing the formation of cytotoxic aggregates [60]–[62]. Of these, the best known are the cytosolic genes, as these include the HSPs, specifically HSP70 which has long been associated with the cellular stress response (CSR) [58].

Chaperones do not act in isolation, efficient protein folding in the cell is often achieved by the cellular equivalent of a production line, with upstream chaperones capturing and transferring elongating, nascent chains to more specialised systems. In the cytosol, there are two main pathways involving either HSP70 or prefoldin [61]. The HSP70 route is non-specific, whilst prefoldin seems to specifically act in conjunction with the chaperonin that contains T-complex polypeptide 1 (CCT) proteins. In the ER, the route is, again, either via an HSP70 gene family member: BiP (binding protein) or calreticulin/calnexin. Numerous components of all of these chaperone pathways are present in the *E. superba* dataset. These will be described below, of which, given our research interests, the HSPs will be characterised in most detail.

### The HSP cytoplasmic chaperones

#### HSP70s

The heat shock proteins are a large family of proteins which are named according to their molecular weight in kiloDaltons. The most studied of these are the 70kD heat shock proteins (HSP70s). There are two main forms of these 70kD proteins, the heat shock cognate (HSC70) which is expressed constitutively and an inducible form (HSP70) which is normally expressed in response to external stimuli [58]. Whilst HSP70 is the “classical” stress gene, the expression levels and regulation of these gene family members in Antarctic species is of particular interest for two reasons: protein folding is more difficult at low temperatures and in the limited examples studied to date, Antarctic species show enhanced expression of these genes [63]–[66] with one adaptation to the cold being the permanent expression of the inducible form in the fish [63], [64], [66] and also the lack of the classical heat shock response (HSR) [65]–[69].

In the krill assembly, 16 contigs, comprising a total of 1,026 reads (coverage ranging from 2–323 reads per contig) produced the best sequence matches to HSP70 using BLAST searching tools. Ten sequences showed the most sequence similarity to the classic inducible form of HSP70. The alignment of the 9 non-redundant sequences (Figure S1) showed that only contig 02253 was complete. The nucleotide sequence of this contig putatively codes for a protein of 668 amino acids. The percentage identity of the deduced amino acid sequence of contig 02253 compared with orthologues from other crustaceans is very high, between 78–84%, confirming the remarkable conservation of this family. As could be expected, this percentage is even higher when you only consider comparisons within the super order *Eucarida* (>81%) or outside the class *Malacostraca* (<79%). More detailed analysis of this full length protein sequence reveals the presence of three signature motifs of the HSP70 family: IDLGTTYSCV (amino acids 9–18), IFDLGGGTFDVSIL (amino acids 196–209), IVLVGGSTRIPKIQKL (amino acids 334–349). Interestingly, at the C-terminus there are four repetitions of a tetrapeptide sequence (GGMP) (Figure S1). Certain of these repetitive peptides have antigenic properties [70] and are also implicated in the association between HSP70 and HSP90 in a multichaperone complex. The ATP/GTP active site is also identifiable via the motif: AEAYLGAT (amino acids 133–138) and a potential bi-partite nuclear targeting sequence KRKHKKDPADNKR at amino acids 252–264. The terminal sequence motif of the HSP70 family identifies their cellular location, with the EEDV sequence motif at the end of contig 02253 attesting to its cytoplasmic localisation [71], whereas HDEL and PEAEYEEAKK characterise members localised to the endoplasmic reticulum and mitochondria respectively.

With regard to the other contigs in the alignment, 19890 and 19889 could potentially be the product of a duplication of HSP70 as the amino acid translations are very similar to that of 02253. The differences between these 3 sequences are possibly explained by the multiple origin of the animals used and therefore constitute allelic variants. A fourth sequence, contig 06573, was relatively short (423bp) and closely resembles the previously described contigs. However, it may represent another form due to the signature situated at amino acid 189 (STGQ) (Figure S1). Whilst this sequence is at the end of the read and therefore the quality is less assured, these supplemental amino acids are also present in the HSPs of other crustaceans. For example, there is a short succession of amino acids intercalated at a similar position (amino acid 191 in the alignment in Figure S2) in the two HSP70 isoforms of the hydrothermal vent shrimp, *Rimicaris exoculata*, as well as in the crab, *Dromia personata* and the oysters *Ostrea edulis* and *Crassostrea gigas* (Figure S2). Whether contig 06573 represents a duplication of the HSP70 gene in *E. superba* is difficult to exactly determine without the full length sequence and extensive PCR amplification between sequence fragments, which is planned in a future study. However, there is evidence of a duplication of HSP70 genes in Antarctic species; in the molluscs *Laternula elliptica*, *Nacella concinna*, but also the crustacean *Paraceradocus gibber* [65], [69], [72] and other crustacean species known by their capacity to respond to thermal shock namely, *Rimicaris exoculata* [73], *Palaemonetes varians* [74] and *Macrobrachium rosenbergii* [75] amongst others.

Of the other contigs represented in the HSP70 alignment (Figure S1), contig 20245 (and 20197, which is not shown in the alignment as it is an exact duplicate sequence of 20245) is clearly different to those described above, although this is not possible in comparisons with 06573 as there is no overlapping sequence. On BLAST sequence similarity searching these two sequences represent the most likely candidates for the constitutive form of HSC70 (HSPA8), but at 531bp and 163bp respectively accurate designation was difficult.

The overall conclusions from examining the contigs listed above, permits the observation that *E. superba* possesses numerous forms of HSP70. The designation of specific isoforms coding for inducible or constitutive expression patterns is a delicate issue, based on sequence alone. Only by following the expression of these variant molecules under different regimes and environmental stresses will enable the true definition of their role.

#### HSP90

Six contigs showed high sequence similarity to HSP90. The full length sequence, with the exception of 4 amino acids from the N-terminal region was constructed from 4 overlapping contigs 00022, 00026, 02405 and 02406 (Figure S3). This sequence of 2151 nucleotides coded for a protein of 717 amino acids containing the characteristic signatures for the HSP90 protein: NKEIFLRELISNSSDALDKIR (amino acids: 28–48), LGTIAKSGT (amino acids: 95–103), IGQFGVGFYSAYLVAD (amino acids: 119–134), IKLYVRRVFI (amino acids: 346–355) and GVVDSEDLPLNISRE (amino acids: 372–386) [76]. The presence of the pentapeptide MEEVD at the extreme C-terminal indicated that this *E. superba* EusHSP90-1 is cytosolic and is also implicated in the binding of known co-chaperone molecules.

Two other contigs showed high sequence similarity with HSP90 (10288 and 00025), but there were sufficient amino acid differences present (Figure S3) to suggest that a second HSP90 gene exists in *E. superba* (designated EusHSP90-2). Two cytoplasmic HSP90s exist in human (HSP90 α and β) and have been reported in other vertebrates. In the invertebrates, only one protein form has been characterised, although in *Mytilus galloprovincialis* it may be the product of either one or two genes [77] and only one form has been cloned in the crustaceans *Peneaus monodon* and *Metapenaeus ensis* [78], [79]. Recently, two different HSP90s have been identified in the crab *Portunus trituberculatus* [80]. Analysis of the two ptHSP90s showed that both were most similar to the vertebrate HSP90α and are the result of a duplication of the orthologous vertebrate HSP90α gene [80]. Comparisons were made between these two crab sequences and the *E. superba* sequences across a common region of 128 amino acids. EusHSP90-1 shows most similarity to the HSP90-1 of *Portunus* (86.7% amino acid identity) (79.7% with ptHSP90-2 and 74.2% with EusHSP90-2). Likewise EusHSP90-2 is also most close in terms of identity to ptHSP90-1 (78.1%) than to the second form (76.6%) of ptHSP90. It should be noted that the percentage identity of the two forms of HSP90 in *Portunus* is 86%.

**Figure 1:**
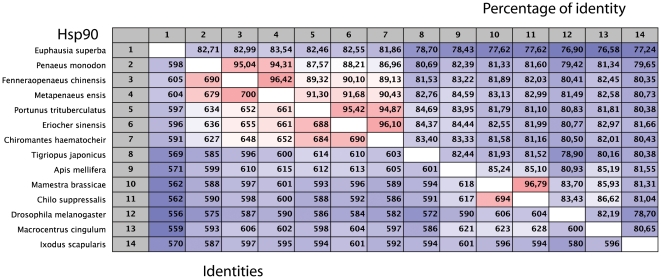
Percentage amino acid identities between HSP90 genes from the *Panarthropoda*. For accession numbers see Figure 2.

**Figure 2:**
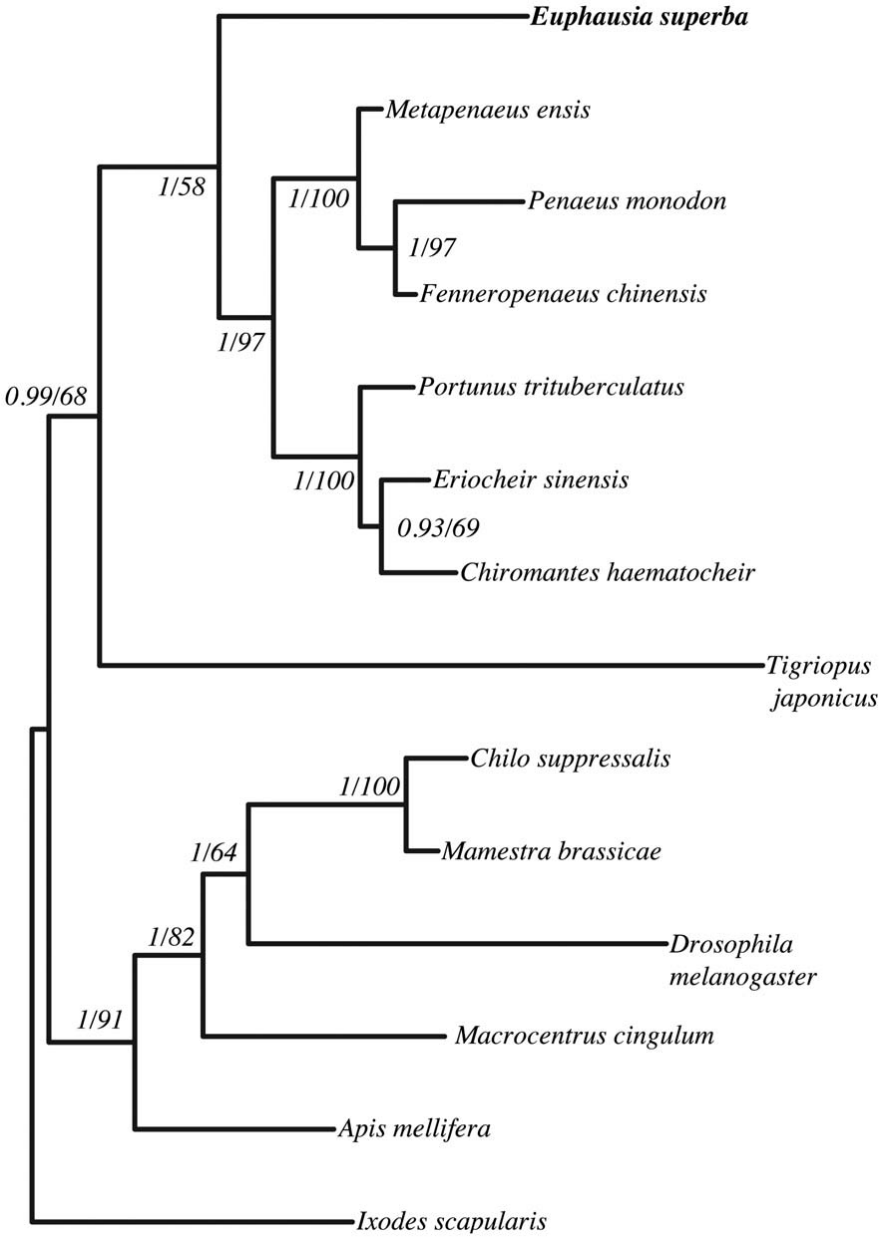
Phylogeny based on Bayesian analysis and maximum likehood of the amino acid data set of HSP90 sequences from the pancrustaceans (14 taxa, 689 characters). *Ixodes scapularis* was assigned as outgroup. Trees obtained by Bayesian analysis and by maximum likelihood analysis of the amino acid dataset were fully congruent. Numbers at nodes are posterior probabilities and bootstrap values (based on 100 replicates) respectively obtained from the analysis of the amino acid dataset. Sequence accession numbers are: *Ixodes scapularis*: XM_002414763; *Apis mellifera*: FJ713701; *Macrocentrus cingulum*: EU570066; *Drosophila melanogaster*: NM_079175; *Mamestra brassicae*: AB251894; *Chilo suppressalis*: AB206477; *Tigriopus japonicus*: EU831278; *Chiromantes haematocheir*: AY528900.1; *Eriocheir sinensis*: EU809924; *Portunus trituberculatus*: FJ392027; *Fenneropenaeus chinensis*: EF032650; *Penaeus monodon*: FJ855436; *Metapenaeus ensis*: EF470246; *Euphausia superba*: contig00022. Only the ptHSP90-1 and EusHSP90-1 are used in this analysis.

These observations lead to the hypothesis that the Crustacean HSP90-1 genes derive from a common ancestor and that the sequences described here constitute orthologues. However, the percentage identities between the different forms of HSP90 underline that both forms of the crustacean gene are closest in terms of sequence similarity with the vertebrate α form. Hence these molecules are the result of an independent duplication event and the high variability between the paralogues observed in the *Euphausia* indicate that this phenomenon was very ancient in this taxa. This hypothesis is in accord with the results observed in the *Peneids*, which at the phylogenetic level are intermediate between the *Euphausia* and the *Brachyura* and only appear to have a single HSP90 gene [78], [79]. The HSP90 sequences from a number of invertebrates belonging to the *Panarthropoda* were aligned (Figure S4) and show the conservation of the protein in this taxon (Figure 1). Bayesian and Maximum Likelihood analyses using the Chelicerate *Ixodes scapularis* as an outgroup generated congruent trees positioning krill as the sister group of the decapods (Figure 2). The resulting tree structure is coherent with regard to the positions of the different taxons [81], [82], even if certain nodes are weakly resolved, i.e. the base of the *Eucrustacea* (Figure 2).

**Figure 3:**
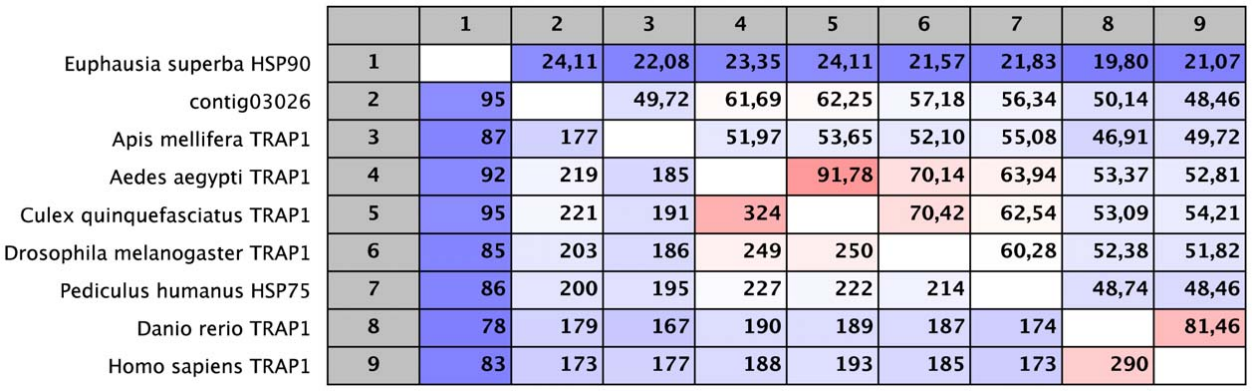
Percentage amino acid identities of TRAP1 sequences. These are from a number of invertebrates and vertebrates with the EusHSP90-1 and contig03026. Accession numbers:. *Aedes aegypti* TRAP1: AAD29307; *Culex quinquefasciatus* TRAP1: XM_001861228; *Drosophila melanogaster* TRAP1: AAD29307; *Pediculus humanus* HSP75: XP_002425720; *Danio rerio* TRAP1: AAI4468; *Apis mellifera* TRAP1: XP_623366; *Homo sapiens* TRAP1: Q12931.

The last sequence with high sequence similarity to HSP90 (contig 03026) putatively codes for the terminal 354 amino acids. However, this shows considerable differences with EusHSP90-1 (24% identity over the region concerned) and does not contain the cytosolic signature EEVD at the extreme C-terminal. Further analysis of the BLAST results indicated that this sequence is actually more similar to TRAP1 (Tumour Necrosis Factor (TNF) Receptor-Associated Protein 1) or HSP75, even though some of the database matches have been designated as HSP90 (*Aedes aegypti* and *Culex quiquifasciatus*). This example demonstrates some of the confusion over the naming of these genes, as in *Homo sapiens*, where all of these variant names have now been potentially designated as HSPC5 [57]. The alignment and percentage identity calculation between the putative *E. superba* sequences and TRAP1/HSP75 genes from different invertebrates and vertebrates showed significant conservation of this molecule in different taxa (Figure 3 and Figure S5). This homologue of the HSP90s is localised in the mitochondria [83], [84]. Although the precise function of these molecules is yet to be determined, they are implicated in cellular stress resistance pathways and therefore are involved in the general functioning of the cell cycle, cellular differentiation and apoptosis. It is evident that the mitochondria play an essential role in cellular homeostasis under stressful conditions as well as the control of intracellular Ca^2+^, energy generation and apoptosis (see also section on o*ther identified chaperones including mitochondrial genes*). Despite their sequence similarity to the HSP90 genes, the TRAP1 family exhibit significant functional differences [83]. They are particularly studied for their role in cancer defence and represent targets of interest in anti-cancer therapies [85].

### The prefoldin cytoplasmic pathway

The other major cytosolic chaperone system involves prefoldin. This chaperone is the first stage in a pathway that largely acts on correct folding and polymerization of the cytoskeletal proteins, actin and tubulin [86]. It is a multimeric protein comprised of 6 subunits; 2 are α-like (designated 3 and 5) and the remainder are β-like (assigned numbers 1, 2, 4 and 6). This protein processes nascent polypeptides and then passes them onto CCT for further processing. This second chaperone is more complex comprising 16 subunits in 2 stacked rings, each with 8 polypeptide subunits: alpha, beta, gamma, delta, epsilon, eta, theta and zeta. All subunits for both complexes were present in our data set with the exception of CCT theta (Table 3).

**Table 3:** Components of the prefoldin cytosolic chaperone system leading to tubulin polymerisation.

Putative gene designation	Contig ID	Length (bp)	No of reads	Description
Prefoldin	02592	544	24	sub-unit 1: *Caligus clemensi* (sea louse) 2.4e^−23^
	14357	325	4	sub-unit 2: C1BJW5: *Osmerus mordax* (rainbow smelt) 7.6e^−23^
	14161	407	7	sub-unit 3: Q5RCG9: *Pongo abelii* (Sumatran orangutan) 7.2e^−31^
	12250	446	15	sub-unit 4: *Oncoryhnchus mykiss* (rainbow trout) 4.3e^−11^
	06004	1045	62	sub-unit 5: *Culex quinquifasciatus* (mosquito) 7.7e^−37^
	01685	499	39	sub-unit 6: *Ixodes scapularis* (blacklegged tick) 1.6e^−20^
Chaperonin that contains T-complex polypeptide 1 (CCT)	19460	1984	484	CCT1:α subunit: C3Y2C5: *Branchiostoma floridae* (Florida lancelet) 4.5e^−210^
	00135	1864	268	CCT2: β subunit: B5X2M8: *Salmo salar* (Atlantic salmon) 1.7e^−187^
	02814	1917	247	CCT3: γ subunit: B0WSS5: *Culex quinquifasciatus* (mosquito) 3.6e^−199^
	00117	1994	388	CCT4: δ subunit: C3XS21: *Branchiostoma floridae* (Florida lancelet) 1.1 e^−192^
	21580	626	278	CCT5: ε subunit: Q68FQ0: *Rattus norvegicus*(rat) 3.3e-^79^ mid portion of gene
	00119	202	149	CCT5: ε subunit: C0HBB4: *Salmo salar* (salmon) 4.2e^−17^ 3′ end of gene
	00120	204	51	CCT5: ε subunit: Identical clone to contig 00120
	04098	1804	349	CCT6: η subunit: Q95V46: *Artemia sanfranciscana* (brine shrimp) 3.5e^−212^
				CCT7: θ subunit: not present
	00149	1785	376	CCT8: ζ subunit: B0W8W8: *Culex quinquifasciatus* (mosquito) 4.0e-202
Tubulin-specific co-factors	03429	1127	19	tbca: B0W943: *Culex quinquifasciatus* (mosquito) 7.4e^−14^
	02234	2032	62	tbcb: Q5E951: *Bos taurus* (bovine) 5.8e-61
				tbcc: not present
	05722	958	8	tbcd: Q5ZI87: *Gallus gallus* (chicken) 7.0e^−53^
	02424	1104	14	tbcd: Q8BYA0: *Mus musculus* (mouse) 5.0e^−40^
	07816	635	4	tbce: Q28EJ7: *Xenopus tropicalis* (pipid frog) 4.6e^−21^

CCT action is not exclusively restricted to actin and tubulin, as it has also been shown to bind approximately 15% of newly synthesized proteins sized between 30–60kDa and so may play a more general role in the cytoplasm [87]. However, as part of its primary role, CCT may pass on the cytoskeletal folding intermediates for further processing and in the case of tubulin, this can lead to the production of microtubules. This is achieved via tubulin cofactors (designated A–E), all of which are also present in our dataset with the exception of subunit c; subunit d is present in the form of 2 non-overlapping clones, hence the dual entry (Table 3). The tubulin co-factors act on α and β-tubulin folding intermediates to generate polymerizable heterodimers for microtubule growth and centrosome generation, but also confer a certain level of stability within these structures and hence directly influence cell division processes as well as providing a chaperone function [88].

Analysis shows that all major elements of the two cytosolic chaperone systems were present in the *E. superba* data set. Contigs 18509 and 13343 showed strong sequence similarity to members of the HSP70 family and are almost certainly molecular chaperones. However a closer analysis and alignment with database entries for HSP70 isoforms 4 and to members of the HSP105/110 (Figure S6), shows that with these partial sequences, it is impossible to precisely identify which group (HSPA14, HSPH1, HSPH2 or HSPH3) these fragments actually belong to. Similarly, contig 06563 showed a match with high probability to HSP70 protein 14 (HSPA14), but with only 34% identity to the mouse protein, it is either not highly conserved between species or is a different, potentially novel family member. Only by comprehensive analysis of all HSP70 family members will the exact designation of vertebrate orthologues be possible in non-model species, a non-trivial task.

Similarly, HSPH1 (contig 18509) is another member of the HSP70 family and whilst it has been shown to prevent the aggregation of proteins under severe stress, it also interacts with other HSPs, inhibiting HSPA8/HSC70 ATPase and chaperone activity. In addition, a number of other cytosolic chaperones and co-chaperones were present, some of which appear to have mainly chaperone functions (e.g. HSPa4l (contig09322)), whilst others interact with, and control the action of other chaperones. Of particular note were contigs with high sequence similarity to the HSP70 co-chaperones DnaJ, chaperonin 10 (contig 01709) and HSPBP1 (contig06138). This latter protein interacts with HSPA1A, an HSP70 family member and has been shown to inhibit the action of HSPA1A and therefore impact on degradation of target proteins. The DnaJ proteins are an extensive gene family which directly stimulate the ATPase activity of the HSP70 chaperones, but also determine the activity of HSP70s by stabilising their interactions with substrate proteins [89]. All members of this gene family contain a J domain, but they have been divided into 3 main groups depending on what other domains are present in the protein. In humans, so far there have been 4 type A, 14 type B and 22 type C DnaJ proteins described [57]. In the *E. superba* dataset 18 contigs potentially comprising at least 13 different family members over all three groups were present (Table 4), indicating a similar complement of these genes in krill compared to vertebrates. Of the full data set, only 2 small heat shock proteins were identified in contigs 05090 and 05374. These are difficult to define as they are not very conserved between species. Finally, three non-overlapping contigs were identified with high sequence similarity to Hop. This is the gene name for the HSC70/HSP90 organising protein and mediates the association between these two chaperones. However, as stated previously, the cytosolic chaperone system with the prevalence of HSP70 gene family members is not the only cellular chaperone system or pathway.

**Table 4:** Putatively identified HSP40 genes in *E. superba*.

Contig ID	Length (bp)	No of reads	Putative assignment	Closest match
04109*	1336	29	Dnaja1	Q0P4H4: *Xenopus tropicalis* (toad) 2.2e^−117^
02240	1358	30	Dnaja3	Q16R72: *Aedes aegypti* (mosquito) 6.9 e^−138^
14139	589	5	Dnaja3	D2A3L8: *Tribolium castaneum* (red flour beetle) 2.0e^−155^
09733	412	5	Dnajb1	Q28F52: *Xenopus tropicalis* (toad) 1.5e^−9^
02096	1169	23	Dnajb11	B0WE51: *Culex quinquefasciatus* (mosquito) 1.6e^−115^
09747	1095	23	Dnajc2	Q6NWJ4: *Danio rerio* (zebrafish) 3.6e^−63^
01898	1254	28	Dnajc3	B7P6D4: *Ixodes scapularis* (tick) 4.1e^−87^
08765	603	5	Dnajc7	Q48AJ9: *Tetraodon nigroviridis* (pufferfish) 1.4e^−61^
13604	454	8	Dnajc7	B6VFL2: *Bombina orientalis* (toad) 4.5e^−28^
11977	1179	11	Dnajc9	Q5EAY1: *Xenopus laevis* (toad) 5.7e^−45^
05667	1086	11	Dnajc11	B7PLX9: *Ixodes scapularis* (tick) 9.4e^−71^
06410	441	3	Dnajc13	D3ZNI6: *Rattus novegicus* (rat) 4.5e^−17^
08550	1726	11	Dnajc16	B7QBO3: *Ixodes scapularis* (tick) 1.5e^−36^
16253	542	5	Dnajc19	A8Q605: *Brugia malayi* (nematode) 1.7e^−13^
00224	632	6	Dnajc21	O62360: *Caenorhabditis elegans* (nematode) 3.1e^−28^
**Fragments less than 400bp**			**16579** (266, 5); **14282** (310, 2); **10787** (399, 4);	

Only contigs with reads above 400bp in length are annotated. * denotes full length sequences. Small fragment data includes size in bp and number of reads (in brackets).

### The endoplasmic reticulum chaperone systems

The ER has its own protein quality control mechanisms and acts, in particular, on secretory proteins. Newly synthesized secreted and membrane-bound proteins are folded and assembled in the ER and failure to achieve correct conformation results in *in situ* tagging, retention and degradation, all still within the ER. This comprehensive quality checking is achieved via two main pathways involving either the HSP70 BiP complex or the calnexin/calreticulin system [90].

### The ER BiP pathway

This system recognises the presence of unfolded regions on proteins containing hydrophobic residues and plays an important role in the folding and post-translational modification of non-glycosylated proteins. BiP is an HSP70 family member, often referred to as GRP78 (HSPA5). Studies involving immunoglobulin binding assays have shown that this protein is part of a multiprotein complex which also includes GRP94 (HSPC4), CaBP1, protein disulphide isomerase (PDI), an HSP40 co-chaperone, cyclophillin B, Erp72, GRP170, UDP-glucosyltransferase and SDF2-L1 [90]. Of the genes in this listing, only BiP and GRP74 were specifically identified in the *E. superba* dataset (Table 5). GRP94 is one of most abundant ER proteins and on sequence similarity is thought to be a homologue of HSP90. An additional number of contigs showed high sequence similarity to members of the PDI family (Table 5), none of which are implicated in the BiP complex described above, but have been shown to localise to the ER and have chaperone activity. They also contain variable numbers of thioredoxin domains, which contribute to the disulphide isomerase activity, involved in the disulphide exchange reactions in post translational modification [91].

**Table 5:** Components of the ER chaperone system.

Gene	Contig ID	Length (bp)	No of reads	Description
BiP (GRP78)	01477	2341	259	B0W934: *Culex quinquifasciatus* (mosquito) 2.7e^−264^
GRP74	01266	1940	41	A5LGG7: *Crassostrea gigas* (oyster) 5.3e^−197^
Calreticulin	00826	1750	502	B8K275: *Fenneropenaeus chinensis* (Fleshy prawn) 1.5e^−152^
Calnexin	08163	1065	16	A5LGG8: *Crassostrea gigas* (oyster) 3.4e^−59^
PDI	00914	1918	456	Erp60: B4QC45: *Drosophila simulans* (fly) 1.1e-^144^. Contains 2 thioredoxin domains.
PDI	05765	1293	53	TXNDC5: Thioredoxin domain containing protein 5: Q8NBS9: *Homo sapiens*(human) 1.9e^−87^. Contains 3 thioredoxin domains.
PDI	01765	2182	124	PDIA6: Q15084: *Homo sapiens* (human) 2.7e^−120^. Contains 2 thioredoxin domains.
PDI	**Fragments less than 400bp: 13243** (352,5); **05245** (344,2); **11325** (330, 4); **13589** (280, 3).			

Small fragment data includes size in bp and number of reads (in brackets).

### The ER Calnexin/Calreticulin pathway

This second pathway affects the folding and post-translational modification of virtually all glycosylated, secreted or integral membrane proteins that pass through the ER. Although calnexin and calreticulin are some of the most abundant chaperones in the ER, they are either not detected, or present in trace amounts in the BiP complex described above. Evidence suggests that these two networks are spatially separated and that as secreted proteins mature they are transported inside the ER from one network (BiP) onto calnexin/calreticulin [90]. These two genes are putatively represented by a full length contig for calreticulin (00826) and a partial 3′ clone (08163) for calnexin. Whilst these two genes share a high degree of amino acid similarity and functionality (Ca^2+^ binding, lectin-like activity and recognition of mis-folded proteins), they have distinct cellular localisations which affect their mode and sphere of action. Calnexin is an integral membrane protein, whilst calreticulin is a luminal protein and thus more mobile. In addition to their role as chaperones, they also play a significant role in ER Ca^2+^ binding and storage. Hence they are also involved in Ca^2+^ homeostasis and ER-dependent Ca^2+^ signalling [92] and therefore are potential candidates for understanding responses to stress both in terms of protein turnover, but also cellular signalling.

### Other identified chaperones including mitochondrial genes

In addition to the various cytosolic chaperones identified, a number of other contigs showed high sequence similarity to more specialised protein folding systems. For example, the MESD chaperone (contig 01700) specifically assists in the folding of β-propeller/EGF molecules within low density lipoprotein receptors (LDLRs) and the nuclear chaperone Asf1 (contig 05542) facilitates histone deposition and histone exchange and removal during nucleosome assembly and disassembly. In contrast, contig13343, in spite of having an N-terminal ATPase domain, has many amino acid differences with the classical HSP70s and to date, most research has indicated a primary role in the mammalian immune response.

Similar to the ER, the mitochondria also has its own chaperone system. Two co-chaperones were putatively identified: HscB (contig 12123) involved in iron-suphur cluster assembly and contig 16412 which showed high sequence similarity to GrpE, the main HSP70 co-chaperone in the mitochondria and equivalent to the cytosolic DnaJ [93]. As regards the main HSP70 family member in this organelle, this is GRP75 (HSPA9) (contig 10241). This protein is not induced by heat, but by other forms of stress e.g. glucose deprivation and oxidative injury. Indeed over expression of GRP75 in cell lines has indicated a primary role in inhibition of ROS accumulation under stressful conditions [94].

This latter example exemplifies the multifunctional role that chaperone proteins play in the cell. Whilst they are documented as being one of the major groups of proteins involved in protein folding and the cellular stress response [95], our understanding of their role is becoming increasingly complex. Not only do they assist in the *de novo* folding of nascent proteins (estimated to be in excess of 20% of all cellular proteins), a requirement that increases under stressful conditions, but also in interactions with signal transduction proteins controlling cell homeostasis, proliferation, differentiation and cell death [96]. Hence they are proposed as important multifunctional hubs in cellular networks [97]. As such, even subtle environmental perturbations are proposed as being able to profoundly affect not only chaperone production, but also the functions of the whole cell network [97]. Whilst all major components of the various chaperone systems were identified in this study, despite extensive searches, transcription factors controlling the expression of many of these were not present in the dataset. Sequences with strong sequence similarity to either the Heat Shock Factor (HSF) or the Hypoxia Inducible Factor (HIF) transcription factors were not identified. The reason for this may simply be timing, in that this data represents a single time point and these transcripts had decayed by the time the RNAs were extracted.

Clearly, with regard to the stress response, some of the important interactions to review will be those with the major antioxidant proteins involved in combating the production of ROS, which directly impact on protein functioning.

### Antioxidants

The examination of these enzymes, in terms of their structure and function is closely allied to our interest in heat shock proteins and the cellular stress response and may be especially important for Antarctic species that have evolved at constant low temperatures for millions of years [72], [98]. Sequence similarity searches were carried out for several of the best characterised members of this functional group with good sequence coverage in our data set. In this respect, catalase, a major antioxidant enzyme previously described in the clam [99] is only represented by a single contig (08109) (match: 6.5e^−22^) matching the very 3′ end of the gene (from amino acids 425 onwards). This small amount of predicted amino acid sequence does not really allow for effective annotation or comparisons with other species.

### Ferritin

In contrast, the full length sequence of a putative ferritin gene is present in contig 00056, which is also one of the more highly expressed sequences in the data set (Table 2). This heteromeric protein comprises 2 subunits: a heavy (H) and a light (L) chain. The heavy chain is ubiquitous and contains the catalytic ferroxidase centre which is responsible for the oxidation of iron and allows iron uptake and release. The light chain is catalytically inactive [100]. The sequence described here is the heavy chain variant, containing the feroxidase motif. In line with previous findings with other crustacean ferritins, the *E. superba* sequence shares more amino acid sequence identity with the human (53.6%) rather than the *Drosophila* heavy chain (35.9% identity at the amino acid level) substantiating the theory of differential evolution of the insect ferritins [101]. This protein is well described as being expressed in response to oxidative stress and hypoxia, but interestingly, it has also be shown to be elevated in response to freezing in the marine snail (*Littorina littorea*) [102]. This may explain the relatively high expression levels in this Antarctic crustacean, although it has also been shown to play an immune role in marine invertebrates [38], [103].

### Glutathione S-transferase (GSTs)

The numbers of these genes present in each species varies widely from 16 in human, through 28 in *Anopheles gambiae*, 37 in *Drosophila* to 48 in *Arabidopsis* [104]–[106]. GSTs have been classified into families, the largest of which is the cytosolic family comprising alpha, mu, omega, pi, sigma, theta and zeta. Other family members include a mitochondrial form (designated kappa), membrane bound microsomal forms and some with restricted distributions such as phi, tau and lambda in plants and epsilon and delta in insects.

In the *E. superba* dataset 20 contigs were identified with high sequence similarity to GSTs. Of these, 3 were most likely to be the related prostaglandin D synthases (00275, 02350 and 02637), with contig 09884 most closely matching the mitochondrial kappa form. The remaining were assigned as cytosolic forms and further designated into putative gene family members, based on sequence similarity searches (Table 6). Of the 10 contigs which contained either full length or almost full-length sequences, it was possible to differentiate 8 genes putatively belonging to the omega, sigma, delta, theta and mu families. Of the latter, 4 contigs showed significant matches against the mu family, but three of these (08776, 10471 and 03844) are potentially allelic variants, sharing approximately 85% identity and 93% amino acid similarity. This is in contrast to contig 06543, which also showed sequence similarity to the mu family, but shared only 64.7% identity to the other putative family members and therefore was designated as a separate gene. Previous examination of the *E. superba* ferritin gene, showed this to be evolutionarily more similar to the vertebrate, than the insect lineage. The reverse is true of the GSTs, with the identification of a putative delta GST. Recently a delta GST has been described in the Chinese mitten crab [107] and the data presented here substantiate these findings that the delta GSTs are also present in the *Crustacea* and not restricted to the more traditional insect taxonomic groupings.

**Table 6:** Putatively identified GST genes in *E. superba*.

Contig ID	Length (bp)	No of reads	Putative assignment	Closest match
02590*	1069	45	delta	A9QUN5: *Blattella germanica* (German cockroach) 7e^−38^
03298	1065	16	omega	B0VZ90: *Culex* quinquifasciatis (Southern house mosquito) 2.6e-^47^. 5′ end missing
07558*	976	14	omega	D1MAK0: *Anopheles cracens* (mosquito) 2.4e^−44^
08955*	950	22	sigma	O18598: *Blattella germanica* (German cockroach) 3.1e^−17^
06543*	848	88	mu	C3KHT9: *Anoplopoma fimbria* (sable fish) 9.2e^−65^
10471*	820	19	mu	B8JIS5: *Danio rerio* (zebrafish) 4.7^e-66^
04853	627	19	sigma	C3V9U8: *Chironomus tetans* (midge) 3.1e^−14^. Mid portion of gene.
03844	603	93	mu	Q0GZP5: *Cyprinus carpio* (common carp) 2.5e^−56^. Partial 5′ end.
08403	531	4	theta	Q5PY76: *Aedes aegypti* (yellow fever mosquito) 7.0e^−33^. Partial 5′ end.
08776	417	7	mu	B8J188: *Danio rerio* (zebrafish) 2.5e-^35^. Partial 5′ end.
**Fragments less than 400bp**			**20423** (400, 6); **09683** (268, 4); **12330** (263, 2); **03758** (243, 8); **10951** (243, 13); **20077** (153, 9)	

Only contigs with reads above 400bp in length are annotated. * denotes full length sequences.

Surveying the potential functionality of these proteins in *E. superba*, there are potential clues from the *Insecta*. The sigma class in mammals function as prostaglandin synthases, but in *Drosophila*, these have been shown to be primarily active in the detoxification of oxidative damage [108]. Also the *Drosophila* delta GSTs are involved in combating oxidative stress and metabolism of endogenously formed lipid peroxidation products [109]. It will be interesting to further characterise the function of these sequences in *E. superba* in the light of lipids as a major energy store for Antarctic marine species and the enhanced levels of dissolved oxygen and the potential for increased ROS damage in Southern Ocean species.

### Superoxide dismutase: a novel duplication event

**Figure 4:**
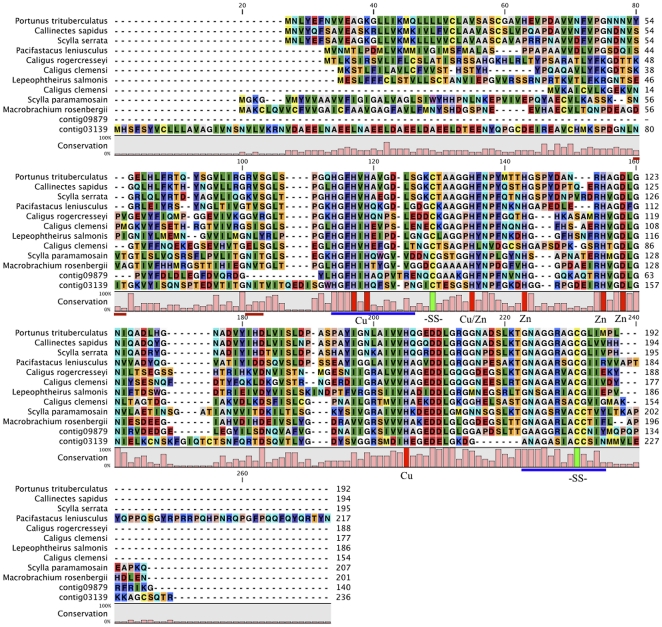
Multiple sequence alignment of the two putative *E. Superba* Cu-Zn superoxide dismutases with those of other *Eucrustacea*. Signature motifs for Cu-Zn superoxide dismutases are indicated by the blue lines under the consensus. The four amino acid residues required for binding copper and four amino acids required for binding zinc are indicated by arrows, as well as two cysteines residues (SS) for the disulphide bridge [110]. Putative N-glycosylation sites for contig03139 only are indicated by the red lines under the consensus and the predicted signal peptide (again for contig03139 only) is marked in italics and underlined. Accession numbers: *Portunus trituberculatus*: C8XTB0; *Callinectes sapidus*: Q9NB64; *Scylla serrata*: A5Y446; *Pacifastacus leniusculus*: Q9XYS0; *Caligus rogercresseyi*: C1BQG7; *Caligus clemensi*: 1: C1C108, 2: C1C2E3; *Lepeophteirus salmonis*: C1BSR9; *Scylla paramamosain*: D2DSH2; *Macrobrachium rosenbergii*: Q45Q33.

Superoxide dismutases are important antioxidant enzymes. Three distinct types have been identified in eukaryotes depending on the metal ions found at the active site: iron SOD (Fe-SOD) (found only in prokaryotes and plants to date), manganese SOD (Mn-SOD) and copper/zinc SOD (Cu,Zn SOD). Surprisingly searches of the *E. superba* dataset identified 4 putative SOD genes (Table 7). Two putative Mn-SOD transcripts were clearly differentiated via the manganese superoxide dismutase signature (DVWHHAYY) and then further defined using BLAST sequence similarity searching into the mitochondrial and cytosolic forms (Table 7). Analysis of the Cu,Zn SODs was more complex. An alignment of the two putative Cu,Zn SOD protein sequences from *E. superba* with those of other *Eucrustacea* identified the conserved amino acid residues for binding Cu and Zn and the signature motifs of Cu,Zn SODs in both genes (Figure 4). There are two forms of this gene documented in a variety of species [110]: an intracellular and an extracellular form [111]. However, the two *E. superba* sequences did not contain either the consensus header or consensus tail sequences (KAVCVL and GVIGT respectively) associated with the intracellular form [110]. Sequence analysis showed that the two *E. superba* transcripts shared only 32.9% identity and hence were not the result of alternative splicing, as has been found in *Caenorhabditis elegans* [112]. Duplicated Cu,Zn SOD variants have been identified in *Xenopus laevis* [113], but the genome of this species is known to be tetraploid and hence this situation may not be unexpected. There is very little data on the genome evolution of the *Eucrustacea*, but, to date, there is no evidence of extra genome duplications in this taxa and therefore the two transcripts in *E. superba* potentially represent a novel species-specific gene duplication.

**Table 7:** Identification of SOD genes via sequence similarity searches.

Contig	Gene	BLAST sequence similarity match					
		Accession number	Organism	Common name	P	Expect	% id
00295	cytMn-SOD	C3W7U6	*Cancer pagurus*	Rock crab	1189	2.3e-119	75
06804	mtMn-SOD	Q3Y596	*Macrobrachium rosenbergii*	Giant fresh water prawn	814	1.2e-79	70
03139	Cu,Zn-SOD	Q09JE3	*Argas monolakensis*	Mono lake bird tick	291	3.3e-24	41
09879	Cu,Zn-SOD	Q45Q33	*Macrobrachium rosenbergii*	Giant fresh water prawn	328	3.9e-28	56

It is not possible, given the data presented here to determine why this gene duplication event, and a requirement for additional antioxidant enzymes, has occurred in *E. superba*. It is suggested that animals living in constant cold temperatures may be more vulnerable to damage by reactive oxygen species, due to slow cell and protein turnover rates and the consequent accumulation of oxidised proteins [99], [114], however examination of the databases shows a complex pattern of Cu,Zn duplication in the *Arthropoda*. *Bombyx mori*, the silk worm, has both intracellular and extracellular Cu,Zn genes. In contrast *Culex quinquefasciatus*, the Southern house mosquito, has 4 different putative genes for Cu,Zn SOD (B0WC98, B0WNS9, B0X9L3, B0VZ56), all of which are different with the latter two containing the intracellular motifs. The two extracellular variants, only show 17.2% identity and 26.9% similarity at the amino acid level, but do contain the required motifs for Cu,Zn SOD, i.e. duplication of both the intra- and extracellular forms. A more complicated story arises with 5 putative genes for SOD in *Drosophila sechellia* (P61854, B4HNH5, B4HMD6, B4ICF4 and B4HT88). The last accession number describes a Mn-SOD, P61854 is the intracellular form of Cu,Zn SOD whilst the other 3 genes (181, 264 and 270 amino acids respectively) all appear to code for different variants of extracellular Cu,Zn SOD. In the *Eucrustacea*, to date, only one form of Cu,Zn SOD has been identified with the exception of the sea louse, *Caligus clemensi*, which has two, but these can be clearly differentiated into an intracellular and extracellular form. In Antarctic marine species, gene duplication events have fuelled adaptations to the cold, particularly in the case of antifreeze proteins [115] and this extra SOD may be another such example. As more non-model species are sequenced the complexity of gene duplication events will become more apparent and fuel the requirement to understand such events in the light of adaptation to different habitats and life histories.

### Other antioxidant genes

Additional to the more detailed analyses above, further contigs were identified with high sequence similarity to genes with antioxidant functions. Contig15252 was shown to be most similar to a copper transport protein in the Southern house mosquito (*Culex quinquifasciatus*) (accession number: B0X8C6). Also two contigs (02344 and 02345) comprised non-overlapping clones with sequence similarity to the ER localised hypoxia up-regulated protein (Hyou1). Whilst this latter protein is a member of the HSP70 family and therefore may play function as a molecular chaperone and participate in protein folding, its critical role is thought to be cryoprotection triggered by oxygen deprivation.

### Conclusions

**Figure 5:**
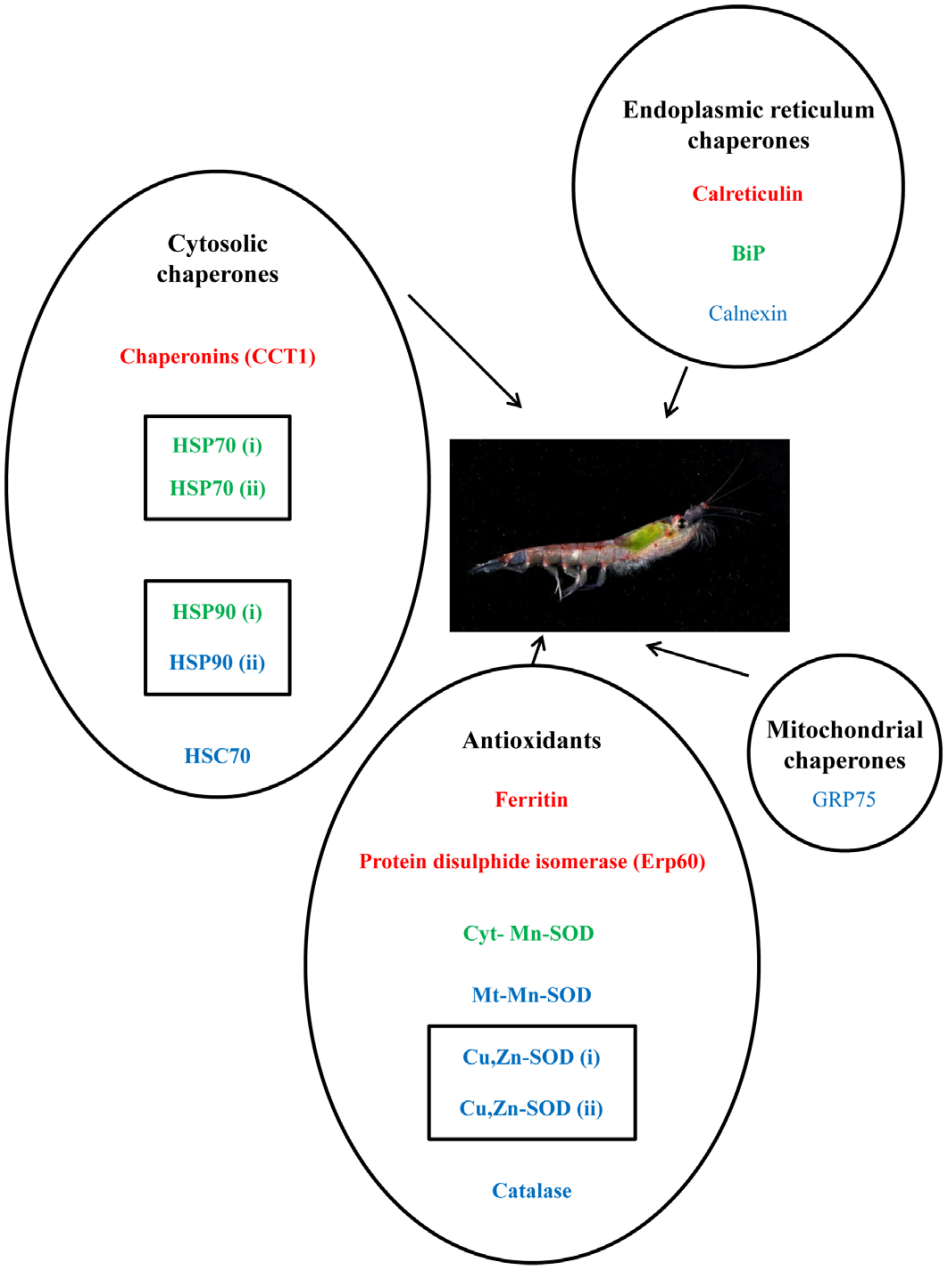
Summary of most highly expressed chaperone and “stress” transcripts in *E. superba*. Picture of *E. superba* from the Bellingshausen Sea continental shelf, taken onboard RRS James Clark Ross cruise JR230 (benthic pelagic coupling cruise) by Pete Bucktrout (British Antarctic Survey). Gene names in red = 400+ copies in the transcriptome data, Green = 200–400 gene copies and blue = less than 200 gene copies. Genes labelled (i) and (ii) plus boxed in the diagram are duplicated genes and their analysis fully described in the main text.

This paper describes, for the first time, a transcriptome of the Antarctic krill *Euphausia superba* using 454 pyrosequencing technologies. The most highly expressed genes are concerned with lipid and seasonal metabolism. However, the classical “stress proteins”, such as HSP70, HSP90, ferritin and GST are also highly expressed, which lead us to carry out an extensive characterisation of both the cellular chaperone system and the major antioxidant proteins (Figure 5). Knowledge of these gene networks is particularly pertinent in the study of Antarctic marine species in terms of both their adaptations to living in an extreme environment, but also to their responses to climate change. In a recent study on the transcriptomes of Antarctic notothenioid fish 177 protein families were shown to be over-expressed compared with temperate relatives, many of these were genes involved in protein biosynthesis, protein folding, lipid metabolism and antioxidants [116], similar to those described in this paper. These were hypothesized as required adaptations to living in the cold and validate some of the proposed costs of living in such an environment, such as decreased protein folding efficiency [117] and increased vulnerability to damage by reactive oxygen species [99].

As more Antarctic transcriptomes become available it will be possible to determine how wide-spread altered expression patterns, gene duplication events and even altered cellular networks are in Southern Ocean or indeed, polar animals. *E. superba* is a pelagic species and experimental work is always limited by availability and ship time. The mere act of recovering the animals from the sea via trawls is stressful and it will always be difficult to partition the stress effects of the sampling regime from the experimental changes to the transcriptome. As such, there is a need to identify not only the stress response, but also the trade-offs associated with enhanced “stress gene” production. It is already known that some Antarctic species permanently express the inducible form of HSP70 as well as other HSP70 family members [63]–[69]. In more experimentally amenable species it has been shown that there is a demonstrable energetic cost associated with the production of heat shock proteins [118] and as yet we have no knowledge of the effect of this in terms of altered transcription patterns in Antarctic species or how it will affect their response to climate change effects. Is the stenothermal nature of Antarctic marine species solely a result of adaptation to living in a cold constant environment for so long, or is it a trade-off effect of survival? Recent large-scale EST and 454 studies [37], [116] and the data described here, are starting to provide the building blocks towards more detailed studies across a range of Antarctic species with the ultimate aim of providing ecosystem level answers to the costs of life in the cold.

## Materials and Methods

### Specimen collection, RNA isolation and cDNA production

The animals were collected on cruise JR177. Catches were made in the vicinity of the South Orkney Islands (60.44°S to 60.53°S and 47.97°W to 48.13°W). All of the krill were taken by target fishing, mainly using a pelagic net (RMT8), based on observations of krill swarms using an EK60 echo sounder at 38 and 120KHz. The nets were towed for only a short time and, after hauling, the krill were transferred as quickly as possible to the cold room at 1–3°C for sorting. Catches were made at different times of the day (00:50, 06:00, 07:00, 14:00 and 20:30) and samples flash frozen before being stored at −80°C. RNA was isolated from whole animals using the RNeasy system with on-column DNase treatment (Qiagen) according to manufacturers instructions. RNA was initially qualified using a standard spectrophotometer and a pool of 6 animals prepared for library production. 1.5µg of pooled sample of krill total RNA was used for the library production, with the RNA purity additionally checked on an Agilent Bioanalyzer. The cDNA library was constructed using the Creator™ SMART™ kit (Clontech Laboratories Inc., Mountain View, CA, USA). In brief, total RNA was reverse transcribed to first stand cDNA coupled with (dC) tailing and CDS III primers (both containing *Sfi*I digestion site sequences) following a step of template switching and extension by reverse transcription. The second strand cDNAs were then amplified using long-distance PCR with Advantage 2 PCR enzyme system (Clontech Laboratories Inc.). The quality of cDNAs was then visually checked by loading the cDNAs on a 1.1% agarose gel. The cDNAs were then purified using QIAquick PCR Purification Kit (Qiagen; Carlsbad, CA, USA) according to the manufacturer's instructions. The second strand cDNAs (5 µg) were then subjected to sequencing library construction and pyrosequencing using GS FLX Titanium system (Roche applied Science, Indianapolis, IN, USA) at McGill University and Genome Quebec Innovation centre (Montreal, QC, Canada) using the standard GS FLX protocol.

### 454 Assembly and Analysis

The initial assembly comprised 943,817 reads. Crossmatch (P. Green, unpublished) was then applied to screen for adaptor sequences and other artifacts of the pyrosequencing procedure and also vector sequences using the UniVec database (www.ncbi.nlm.nih.gov/VecScreen/UniVec.html). Stripping the masked sequence from the ends and removing reads with masked sequence in the middle as well as eliminating resulting reads below 50bp, resulted in 699,248 sequences that were entered into the Newbler program [34] for assembly. This resulted in 22,177 contigs. None of the singletons were used for further analysis. Files containing the reads have been submitted to the National Center for Biotechnology Information Short Read Archive (accession number: SRA023520). The mapping facility of Newbler was applied to the assembly to determine the number of SNPs, and Phobos [119] was used for microsatellite discovery. The contigs were then searched for sequence similarity using BLAST [120] against the genbank non-redundant database [121]. Sequence manipulation was carried out using either the EMBOSS suite of programmes [122] with clustering using ClustalW [123] or CLC Mainworkbench [124]. Alignments were produced and displayed in CLC Mainworkbench [124]. Putative N-glycosylation sites in the Cu,ZN SODs were predicted using the NetGlyc 1.0 Server [125] and signal peptide using the SignalP 3.0 server [126] using both neural networks and hidden Markov models [127] and accessed via the CBS server [128].

## Supporting Information

Figure S1 Putative translations and amino acid alignment of the 9 *E. superba* contigs with sequence similarity to HSP70.
(PDF)

Figure S2 Alignment of the deduced amino acid sequences of the *E. superba* contigs 02253 (full length) and 06573 (partial) with those of other *Eucrustacea*. Sequence accession numbers are: *Balanus amphitrite*: Q86MC3; *Artemia sanfranciscana*: Q95V47; *Moina macropoda*: ACB11341; *Daphnia magna*: ACB11340; *Tigriopus japonicus*: B8PT12; *Pachygrapsus marmoratus*: ABA02164; *Homarus americanus*: ABA02165; *Penaeus monodon*: AAQ05768; *Metapenaeus ensis*: Q1HGN3; *Litopenaeus vannamei*: AAT46566; *Rimicaris exoculata*-3: D2SPE2; *Rimicaris exoculata*-2: ACL52279; *Rimicaris exoculata*-1: ABF85673; *Palaemonetes varians*: ACR77532; *Mirocaris fortunata*: A1XQQ5; *Macrobrachium rosenbergii*: Q6S4R6; *Portunus trituberculatus*: D2DWR3; *Portunus sanguinolentus*: A8KCI1; *Eriocher sinensis*: B5AMI7; *Calinectes sapidus*: Q194W6; *Scylla paramamosain*: B3VKG9.
(PDF)

Figure S3 Putative translations and amino acid alignment of the 6 *E. superba* contigs with sequence similarity to HSP90.
(PDF)

Figure S4 Amino acid alignment of HSP90 genes from the *Panarthropoda*. Sequence accession numbers are: *Ixodes scapularis*: XM_002414763; *Apis mellifera*: FJ713701; *Macrocentrus cingulum*: EU570066; *Drosophila melanogaster*: NM_079175; *Mamestra brassicae*: AB251894; *Chilo suppressalis*: AB206477; *Tigriopus japonicus*: EU831278; *Chiromantes haematocheir*: AY528900.1; *Eriocheir sinensis*: EU809924; *Portunus trituberculatus*: FJ392027; *Fenneropenaeus chinensis*: EF032650; *Penaeus monodon*: FJ855436; *Metapenaeus ensis*: EF470246; *Euphausia superba*: contig00022. Only the ptHSP90-1 and EusHSP90-1 are used in this alignment.
(PDF)

Figure S5 **Amino acid alignment of TRAP1 sequences.** These are from a number of invertebrates and vertebrates with the EusHSP90-1 and contig03026. Sequence accession numbers are: *Aedes aegypti* TRAP1: AAD29307; *Culex quinquefasciatus* TRAP1: XM_001861228; *Drosophila melanogaster* TRAP1: AAD29307; *Pediculus humanus* HSP75: XP_002425720; *Danio rerio* TRAP1: AAI4468; *Apis mellifera* TRAP1: XP_623366; *Homo sapiens* TRAP1: Q12931.
(PDF)

Figure S6 Amino acid alignment of *E. superba* additional putative HSP70 family members with HSP105 and APG HSP70 family members. Accession numbers: *Tigriopus japonicus* HSP105: ACA03526; *Apis mellifera* HSP70Cb: XP_623199.1; *Nasonnia vitripennis*: XP_001607146; *Pediculus humanus* HSP105: XP_002431004; *Xenopus laevis* HSP105: NP_001086692; *Callithrix jacchus* HSP105-2: XP_002748999; *Callithrix jacchus* HSP70-4: XP_002744640; *Mus musculus* HSP70-4-APG2: Q61316; *Mus musculus* HSP70-4-APG1: P48722; *Homo sapiens* HSP70-4: ABM69040.
(PDF)

Table S1 Microsatellite repeats found in excess of 7 copies per repeat unit in *E. superba* data.
(XLS)

Table S2 Variant nucleotides (SNPs/INDELS) found in *E.superba* data.
(XLS)
