# Supplementary figures and images for: Antarctic Krill 454 Pyrosequencing Reveals Chaperone and Stress Transcriptome
Source: PLoS One. 2011 Jan 6;6(1):e15919. doi: 10.1371/journal.pone.0015919 (PMC3017093; doi:10.1371/journal.pone.0015919)

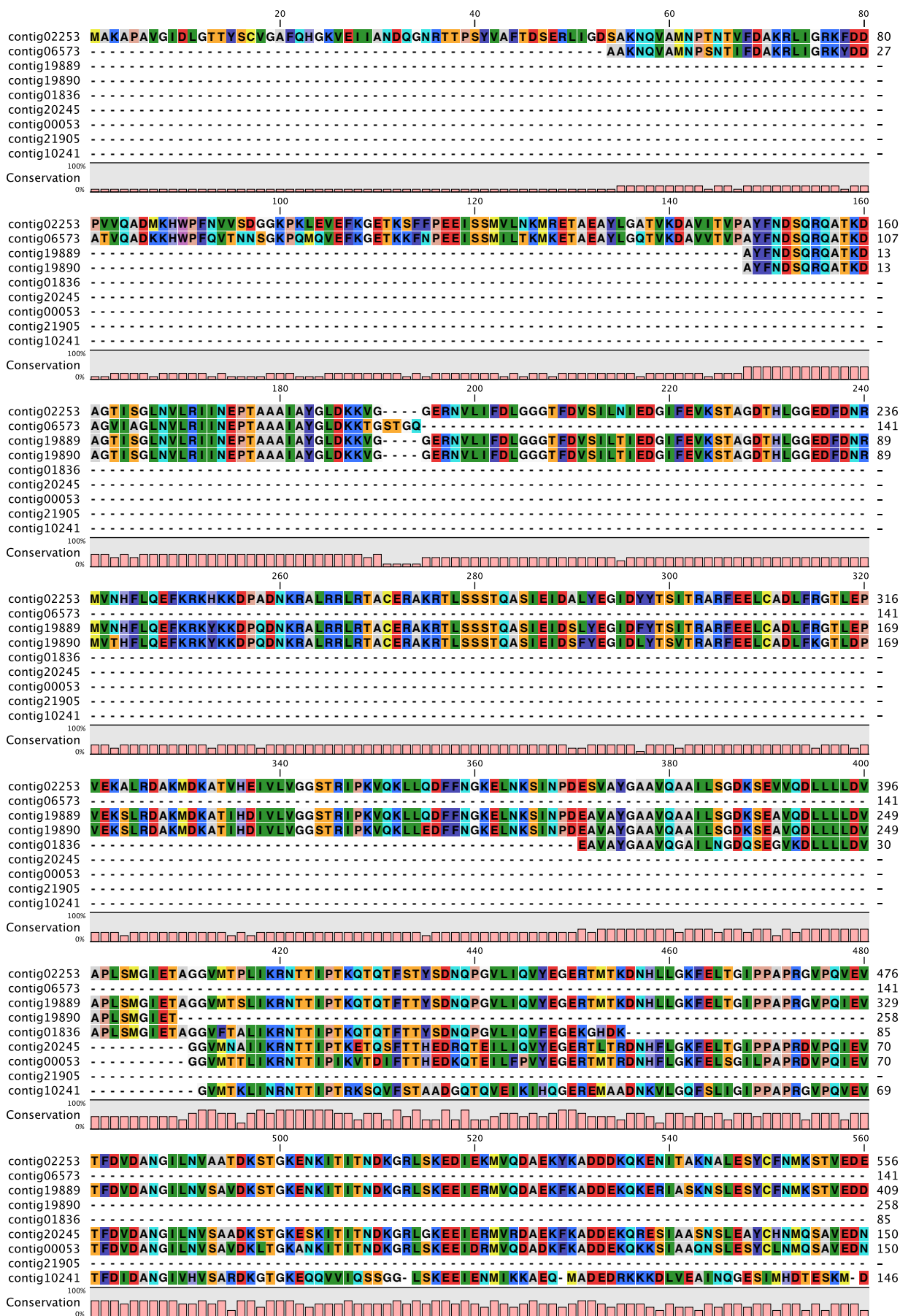

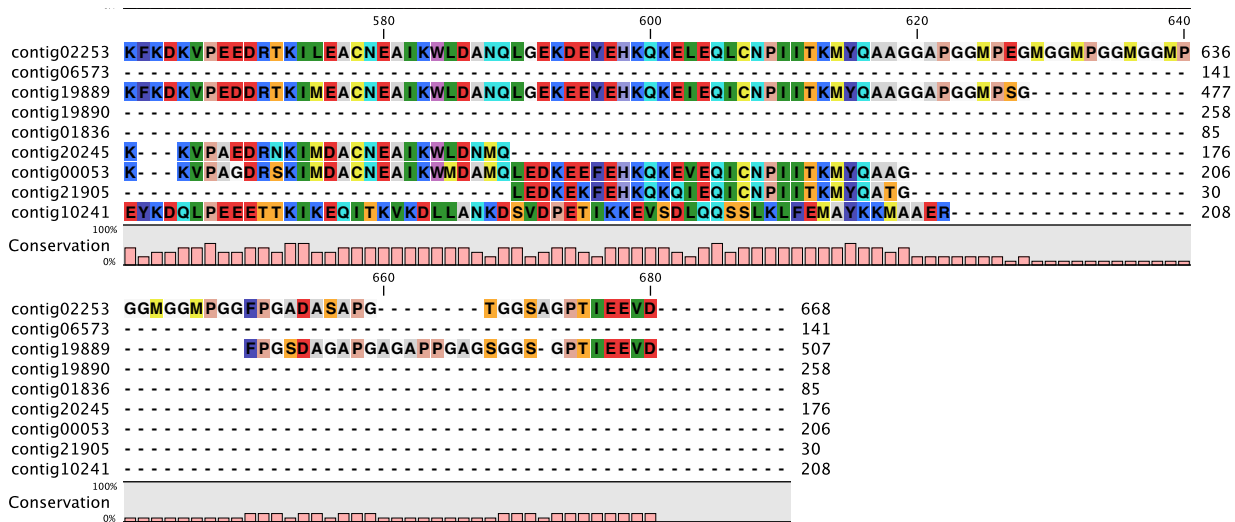

Supplement: Figure S1 — Putative translations and amino acid alignment of the 9 E. superba contigs with sequence similarity to HSP70. (PDF) [file pone.0015919.s001.pdf]

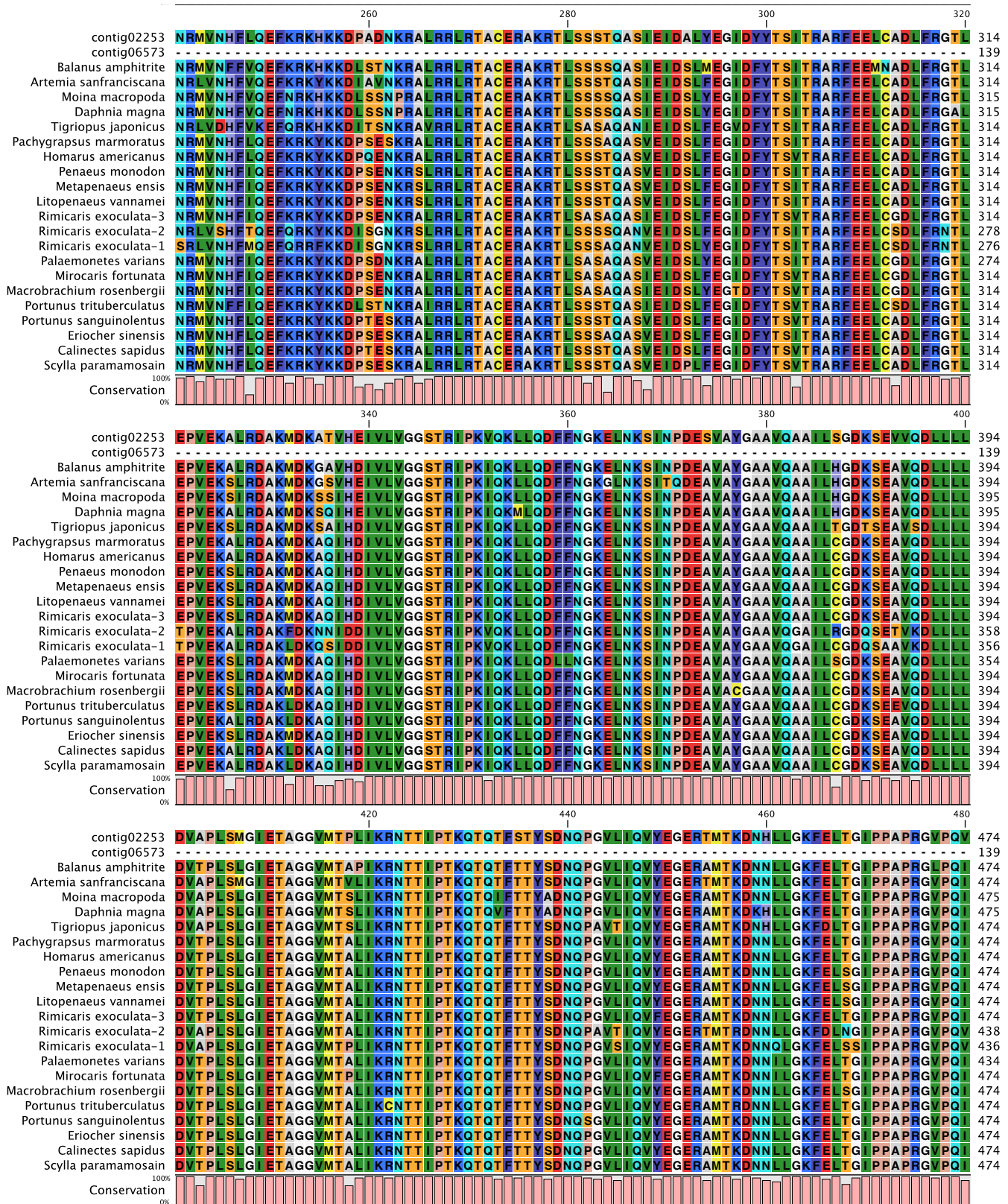

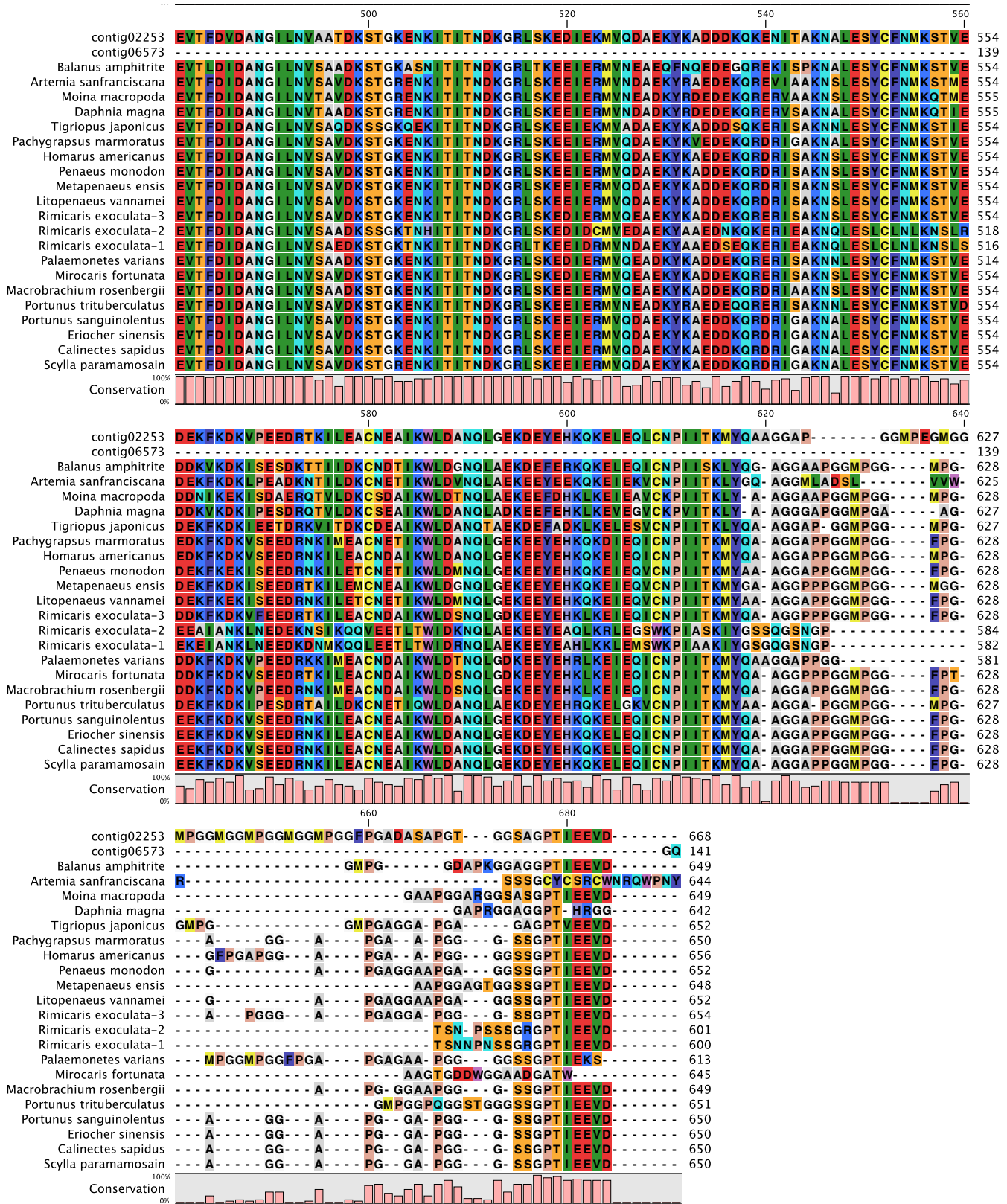

Supplement: Figure S2 — Alignment of the deduced amino acid sequences of the E. superba contigs 02253 (full length) and 06573 (partial) with those of other Eucrustacea. Sequence accession numbers are: Balanus amphitrite: Q86MC3; Artemia sanfranciscana: Q95V47; Moina macropoda: ACB11341; Daphnia magna: ACB11340; Tigriopus japonicus: B8PT12; Pachygrapsus marmoratus: ABA02164; Homarus americanus: ABA02165; Penaeus monodon: AAQ05768; Metapenaeus ensis: Q1HGN3; Litopenaeus vannamei: AAT46566; Rimicaris exoculata-3: D2SPE2; Rimicaris exoculata-2: ACL52279; Rimicaris exoculata-1: ABF85673; Palaemonetes varians: ACR77532; Mirocaris fortunata: A1XQQ5; Macrobrachium rosenbergii: Q6S4R6; Portunus trituberculatus: D2DWR3; Portunus sanguinolentus: A8KCI1; Eriocher sinensis: B5AMI7; Calinectes sapidus: Q194W6; Scylla paramamosain: B3VKG9. (PDF) [file pone.0015919.s002.pdf]

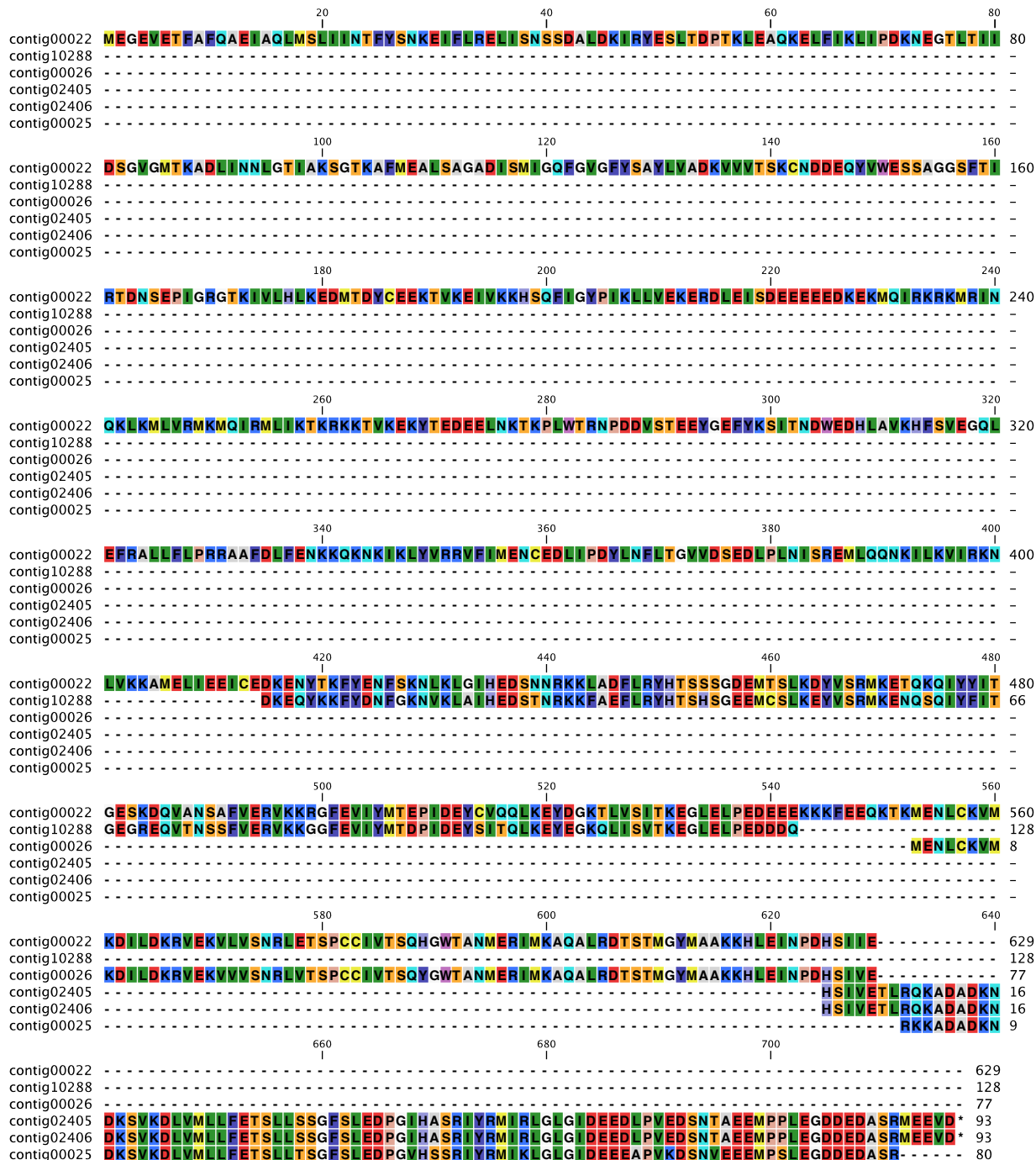

Supplement: Figure S3 — Putative translations and amino acid alignment of the 6 E. superba contigs with sequence similarity to HSP90. (PDF) [file pone.0015919.s003.pdf]

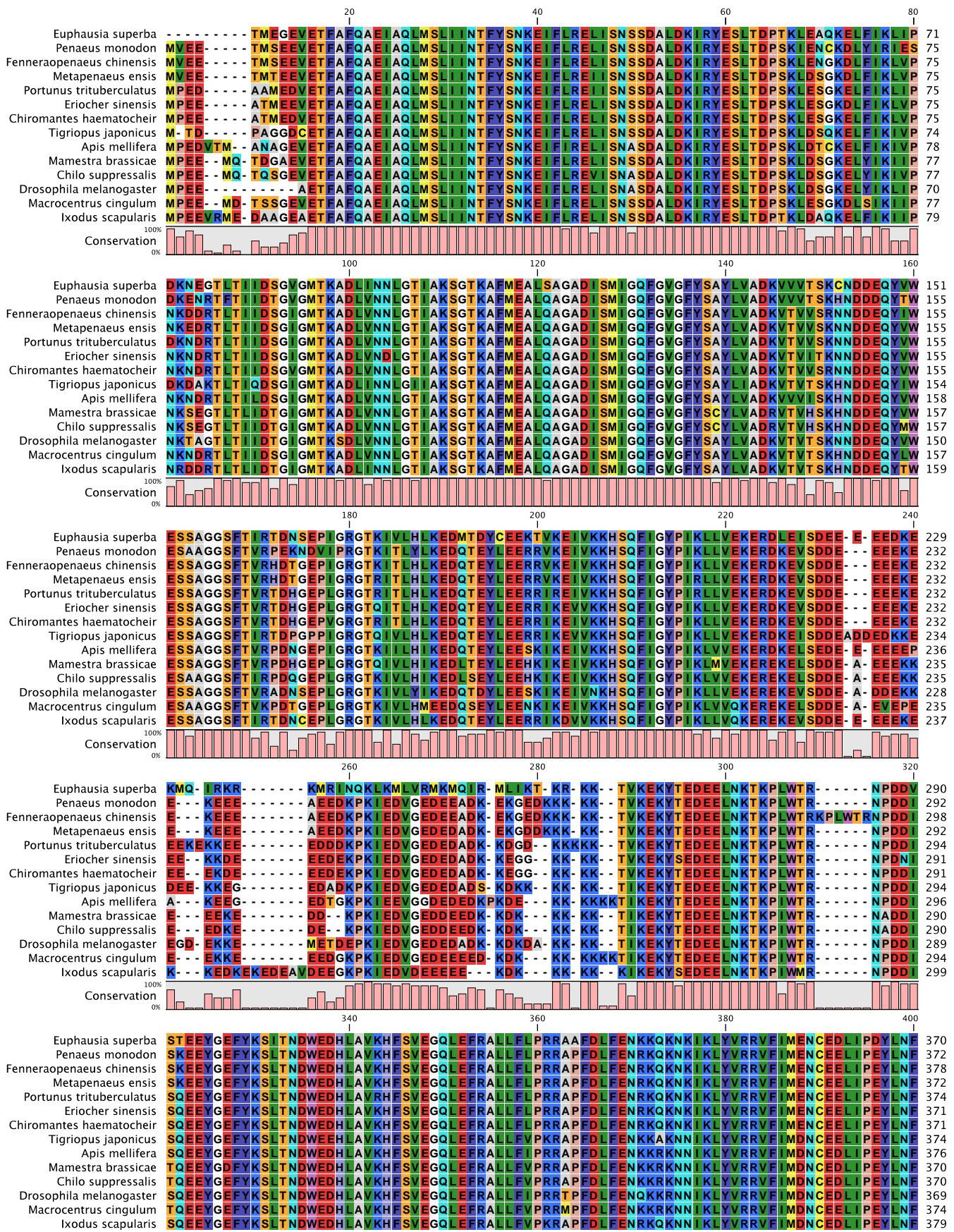

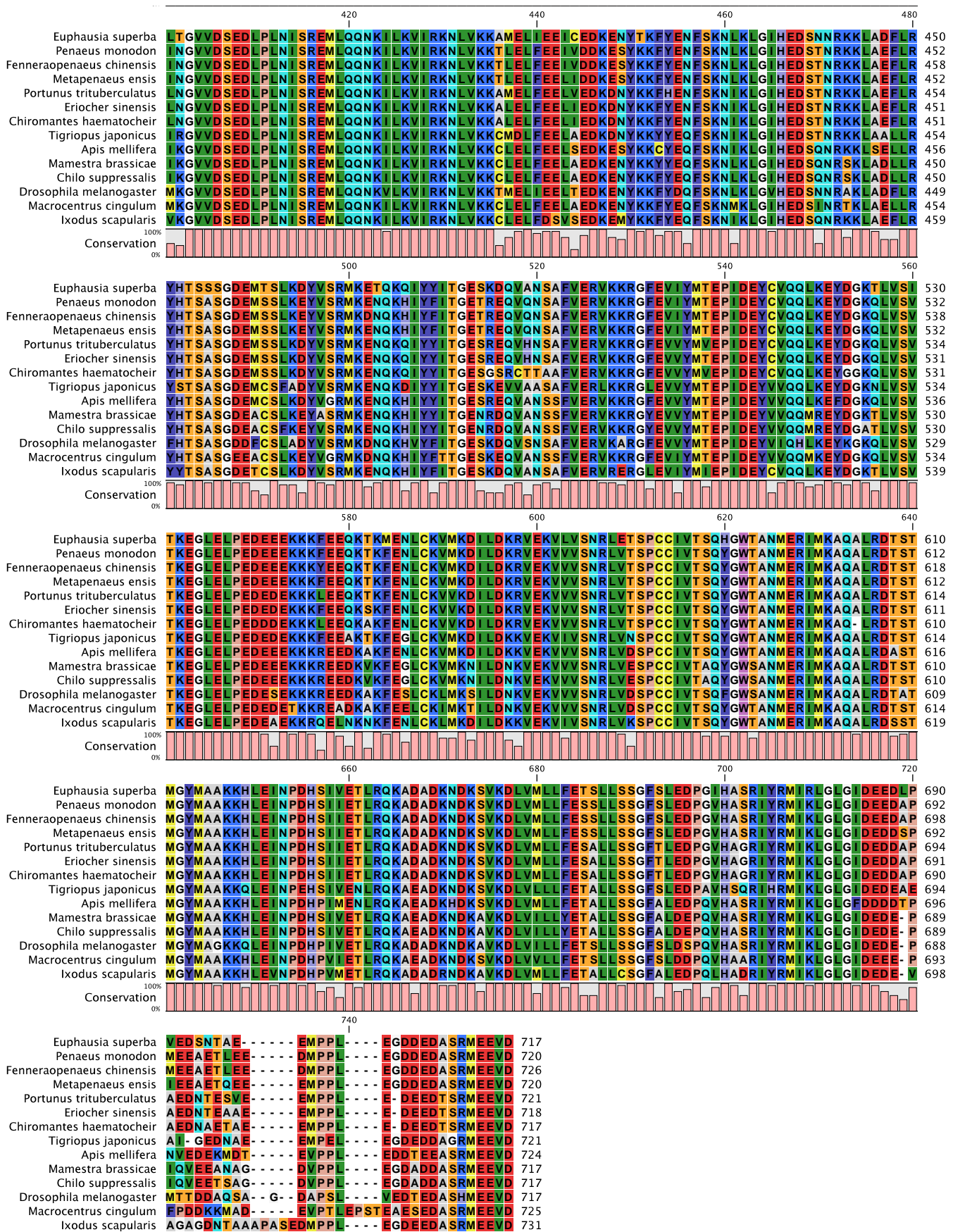

Supplement: Figure S4 — Amino acid alignment of HSP90 genes from the Panarthropoda. Sequence accession numbers are: Ixodes scapularis: XM_002414763; Apis mellifera: FJ713701; Macrocentrus cingulum: EU570066; Drosophila melanogaster: NM_079175; Mamestra brassicae: AB251894; Chilo suppressalis: AB206477; Tigriopus japonicus: EU831278; Chiromantes haematocheir: AY528900.1; Eriocheir sinensis: EU809924; Portunus trituberculatus: FJ392027; Fenneropenaeus chinensis: EF032650; Penaeus monodon: FJ855436; Metapenaeus ensis: EF470246; Euphausia superba: contig00022. Only the ptHSP90-1 and EusHSP90-1 are used in this alignment. (PDF) [file pone.0015919.s004.pdf]

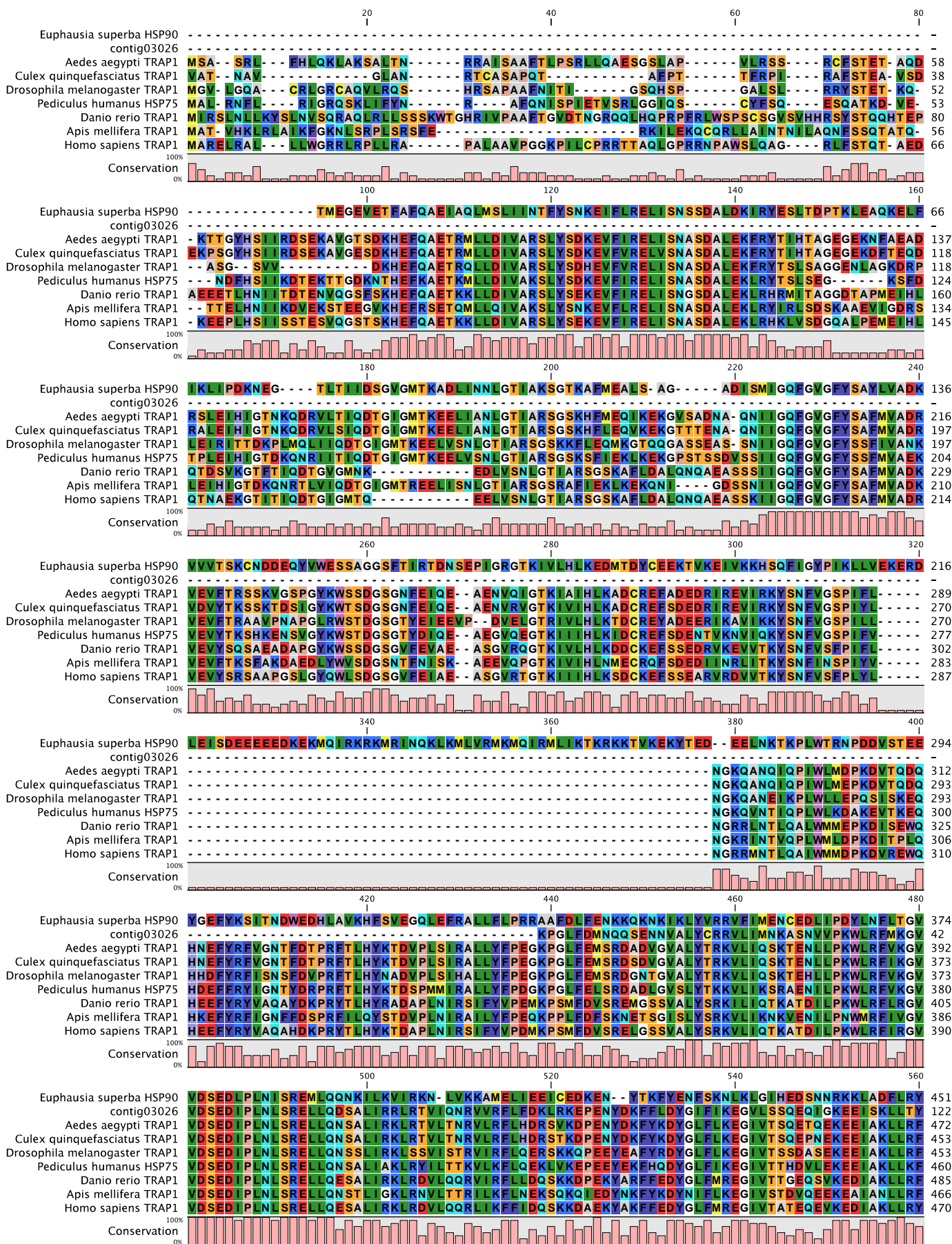

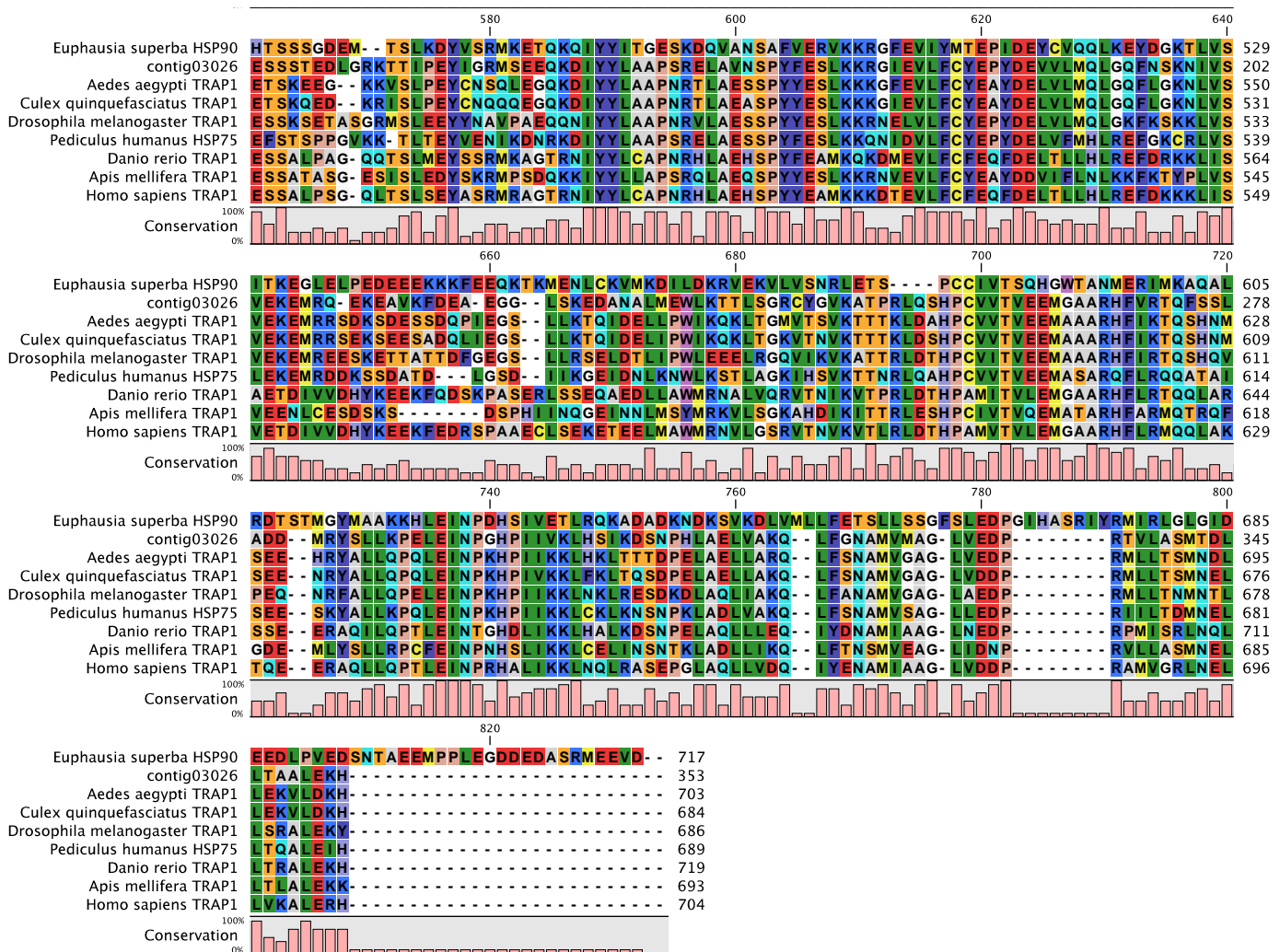

Supplement: Figure S5 — Amino acid alignment of TRAP1 sequences. These are from a number of invertebrates and vertebrates with the EusHSP90-1 and contig03026. Sequence accession numbers are: Aedes aegypti TRAP1: AAD29307; Culex quinquefasciatus TRAP1: XM_001861228; Drosophila melanogaster TRAP1: AAD29307; Pediculus humanus HSP75: XP_002425720; Danio rerio TRAP1: AAI4468; Apis mellifera TRAP1: XP_623366; Homo sapiens TRAP1: Q12931. (PDF) [file pone.0015919.s005.pdf]

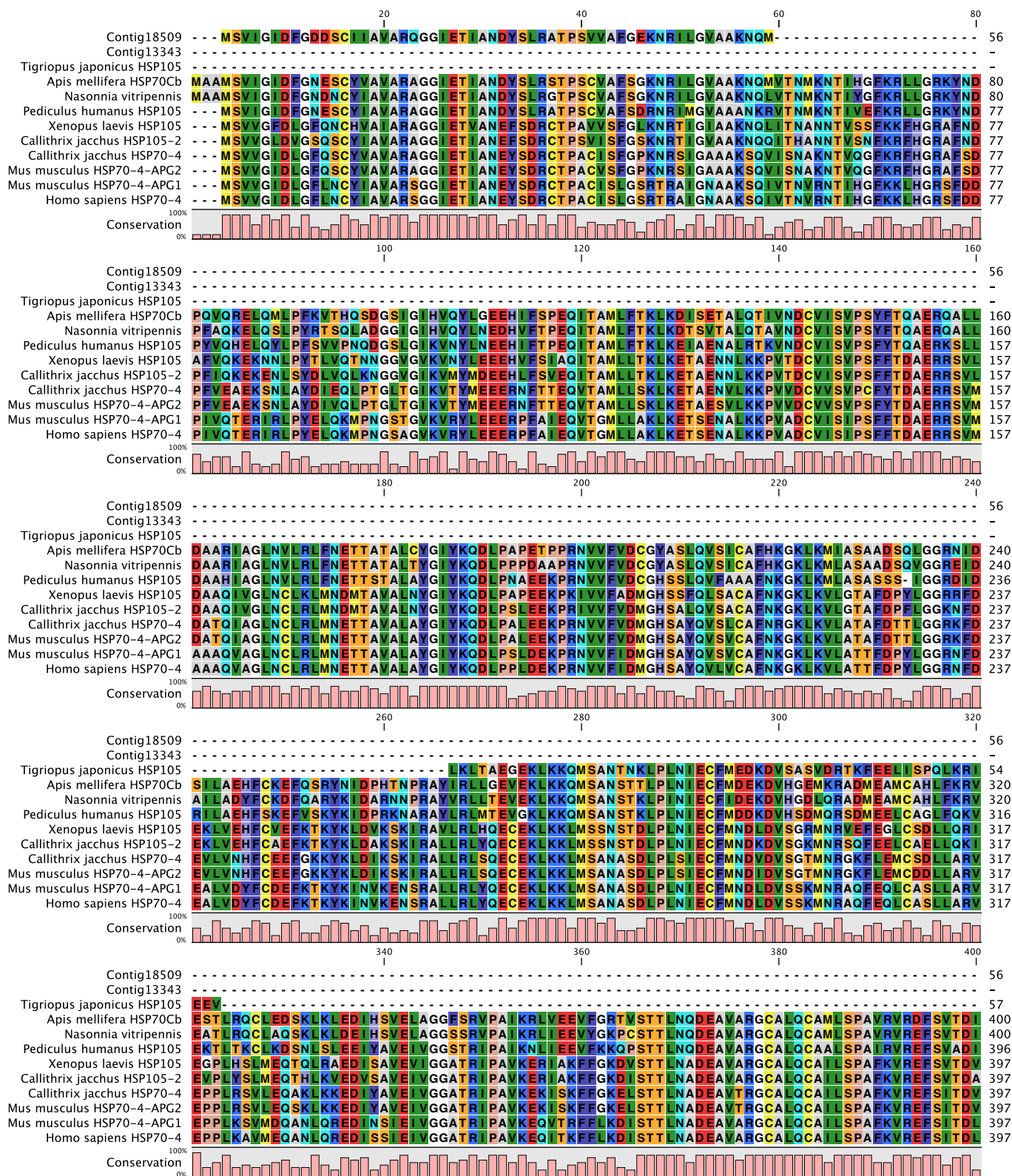

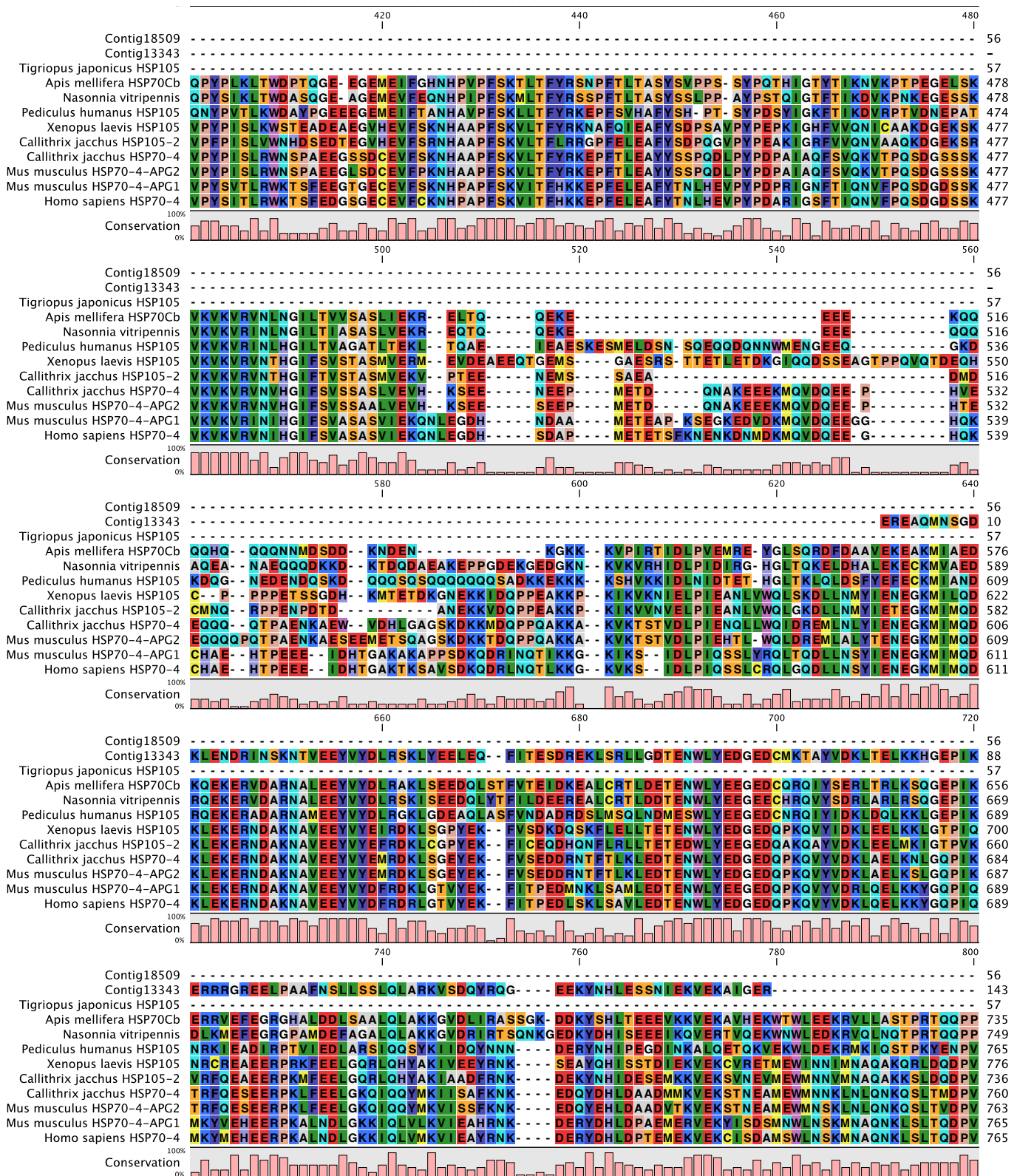

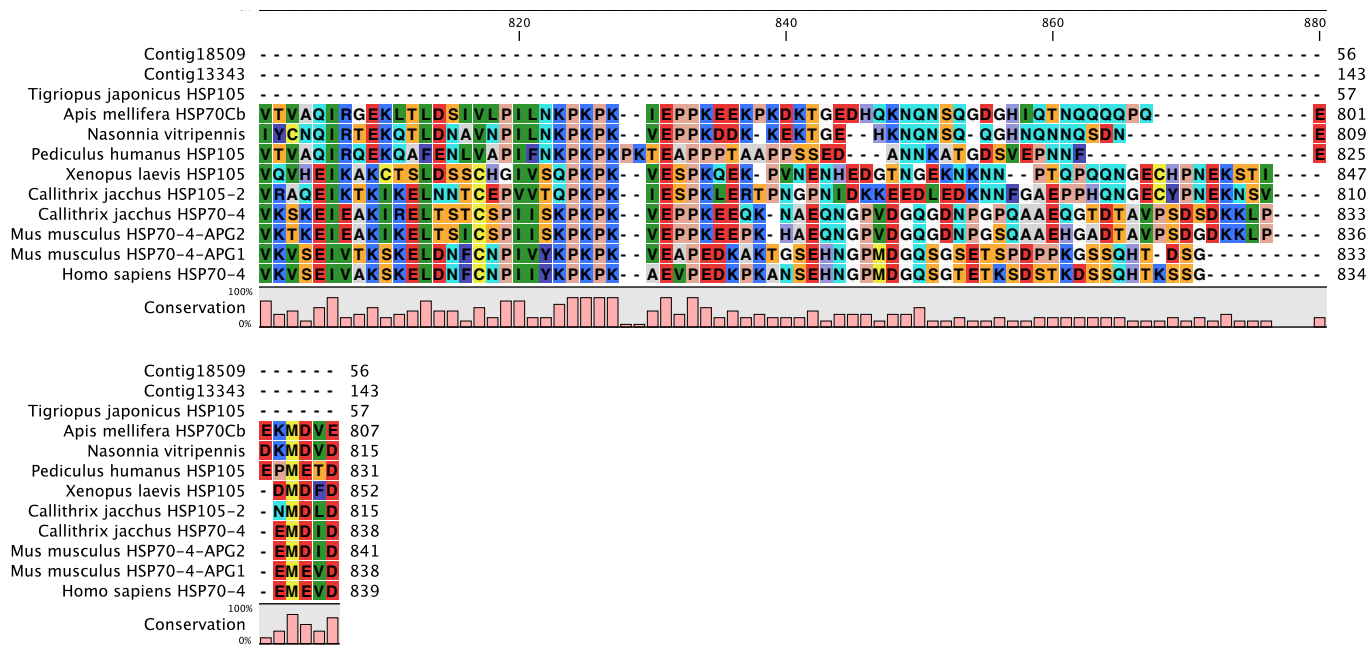

Supplement: Figure S6 — Amino acid alignment of E. superba additional putative HSP70 family members with HSP105 and APG HSP70 family members. Accession numbers: Tigriopus japonicus HSP105: ACA03526; Apis mellifera HSP70Cb: XP_623199.1; Nasonnia vitripennis: XP_001607146; Pediculus humanus HSP105: XP_002431004; Xenopus laevis HSP105: NP_001086692; Callithrix jacchus HSP105-2: XP_002748999; Callithrix jacchus HSP70-4: XP_002744640; Mus musculus HSP70-4-APG2: Q61316; Mus musculus HSP70-4-APG1: P48722; Homo sapiens HSP70-4: ABM69040. (PDF) [file pone.0015919.s006.pdf]
